# Production, Quality Control, Stability and Pharmacotoxicity of a Malaria Vaccine Comprising Three Highly Similar PfAMA1 Protein Molecules to Overcome Antigenic Variation

**DOI:** 10.1371/journal.pone.0164053

**Published:** 2016-10-03

**Authors:** Bart W. Faber, Stephan Hellwig, Sophie Houard, Nicolas Havelange, Jürgen Drossard, Hubert Mertens, Alexander Croon, Robin Kastilan, Richard Byrne, Nicole van der Werff, Marjolein van der Eijk, Alan W. Thomas, Clemens H. M. Kocken, Edmond J. Remarque

**Affiliations:** 1 Department of Parasitology, Biomedical Primate Research Centre, Rijswijk, The Netherlands; 2 Fraunhofer IME, Aachen, Germany; 3 European Vaccine Initiative, Universitätsklinikum Heidelberg, Heidelberg, Germany; 4 Nova Laboratories Ltd., Martin House, Gloucester Crescent, Wigston, Leicester, United Kingdom; Universidad Nacional de la Plata, ARGENTINA

## Abstract

*Plasmodium falciparum* apical membrane antigen 1 (PfAMA1) is a leading asexual blood stage vaccine candidate for malaria. In preparation for clinical trials, three Diversity Covering (DiCo) PfAMA1 ectodomain proteins, designed to overcome the intrinsic polymorphism that is present in PfAMA1, were produced under Good Manufacturing Practice (GMP) in *Pichia pastoris*. Using identical methodology, the 3 strains were cultivated in 70-L scale fed-batch fermentations and PfAMA1-DiCos were purified by two chromatography steps, an ultrafiltration/diafiltration procedure and size exclusion chromatography, resulting in highly pure (>95%) PfAMA1-DiCo1, PfAMA1 DiCo2 and PfAMA1 DiCo3, with final yields of 1.8, 1.9 and 1.3 gram, respectively. N-terminal determinations showed that approximately 50% of each of the proteins lost 12 residues from their N-terminus, in accordance with SDS-PAGE (2 main bands) and MS-data. Under reducing conditions a site of limited proteolytic cleavage within a disulphide bonded region became evident. The three proteins quantitatively bound to the mAb 4G2 that recognizes a conformational epitope, suggesting proper folding of the proteins. The lyophilized Drug Product (1:1:1 mixture of PfAMA1-DiCo1, DiCo2, DiCo3) fulfilled all pre-set release criteria (appearance, dissolution rate, identity, purity, protein content, moisture content, sub-visible particles, immuno-potency (after reconstitution with adjuvant), abnormal toxicity, sterility and endotoxin), was stable in accelerated and real-time stability studies at -20°C for over 24 months. When formulated with adjuvants selected for clinical phase I evaluation, the Drug Product did not show adverse effect in a repeated-dose toxicity study in rabbits. The Drug Product has entered a phase Ia/Ib clinical trial.

## Introduction

The battle against malaria has been going on for centuries and although since half way through the last century the parasite has been eradicated from large parts of the world, there are indications that this progress has come to a halt, despite huge efforts of many companies, governmental and non-governmental organizations and individual people, that have, at the same time, led to a strong decrease in malaria mortality and morbidity [1]. Global warming and concurrent mosquito vectors is deemed responsible for the reintroduction of malaria in formerly malaria-free areas [2].

A key role in overcoming this impasse is reserved for a highly efficacious vaccine against malaria. With the phase III clinical evaluation of the RTS,S vaccine [3, 4] and the promising attenuated sporozoite vaccines [5, 6] considerable progress has been made in this area which will hopefully lead to the powerful tools that are needed in the combat against malaria.

Still, with an efficacy of approximately 30% and “protection against clinical and severe disease”, instead of the anticipated sterile protection, there is room for improvement for the RTS,S vaccine [3]. Moreover, the efficacy in infants wanes quickly, with time and with increasing exposure [7].

With this in mind, we believe that the development of a blood stage vaccine, protecting against the clinical symptoms of malaria will be an essential addition to a pre-erythrocytic vaccine that may be licensed in the near future.

Our blood stage malaria vaccine candidate of choice is apical membrane antigen 1 (AMA1). The immune potential of AMA1 has been reviewed in 2008 [8]. Immunization with AMA1 confers protection in small animals, as well as in nonhuman primates [9, 10]. In a macaque study, it was shown that protection correlated with antibody titres and with *in vitro* growth inhibition activity [10].

A recent phase IIb clinical trial with PfAMA1 from the 3D7 strain in pre-exposed children showed that the infection rate with *P*. *falciparum* 3D7 was significantly lower in the vaccines compared to the control group, hinting to a protective efficacy of 64%, while the overall protectivity was 17% [11]. By contrast, homologous challenge experiments of vaccinated volunteers show little or no efficacy after vaccination with AMA1 [12]. As the observed difference in efficacy between the field and the CHMI challenge in the clinic probably relates to the pre-exposure of the African children to malaria, adaptation of the vaccination protocol in number and/or timing of the vaccination may therefore be an interesting possibility to improve the efficacy of AMA1 based vaccines, exemplified by the protective macaque study [10]. Another interesting possibility to improve the efficacy of an AMA1-based vaccine was illustrated by Srinivasan and co-workers who immunized mice with a combination of AMA1 with a RON2-derived peptide resulting in protection [13]

One of the most important drawbacks of AMA1 as vaccine candidate is its polymorphic nature. Over 20% of its amino acids can change without obvious effects on its function. This has prompted us to not pursue our single allele PfAMA1 vaccine beyond Phase I clinical evaluation [14], but rather develop a vaccine that takes the polymorphism into account. This so-called ‘Diversity-Covering (DiCo)’ approach, comprised of three *in silico* designed PfAMA1 molecules, has been shown to significantly improve the breadth of the humoral immune response, as measured by ELISA and *in vitro* inhibition assays [15].

Here we report on the GMP production, quality assurance, potency and stability of a potential vaccine comprised of equal amounts of the three PfAMA1-DiCo proteins, DiCo1, DiCo2 and DiCo3 that in now used in a clinical phase Ia/b started early 2014 (ClinicalTrials.gov Identifier: NCT02014727).

We will discuss the challenges of the production of the PfAMA1-DiCo proteins, the hurdles that were taken during the development of the production process and the down-stream-processing. Special attention will be given to the development of quality control assays as these are especially challenging due to the high similarity of the proteins.

## Material and Methods

### Construction and selection of expressing clones

Pichia pastoris codon-optimised *pfama1 dico* genes were synthesized by DNA2.0, Menlo Park, CA. The genes differ from the previously described *pfama1 dico* genes [15] with respect to the presence of the prodomain [aa 25–96] of PfAMA1 FVO (Genbank accession no. AJ277646) and a mutation in the domain 2 region (K376→R), that was found to abrogate proteolytic attack in PfAMA1 FVO strain (B.W. Faber, unpublished data). The genes were cloned into the pPicZalpha A vector and transformed to *Pichia pastoris* KM71H to generate Mut^S^ clones secreting PfAMA1-DiCo1, DiCo2 and DiCo3, aa 25 to 545, respectively. Numbering for amino acid positions in the expressed protein is quoted relative to published PfAMA1 sequences, that is without reference to the two N-terminal, vector-derived amino acids expected to be present in the final expressed protein product. Six potential N-glycosylation sites were replaced by an amino acid that is present in other malaria species, as described before [15, 16].

From the lab strains, research cell banks were prepared at IME Fraunhofer. Aliquots thereof were used to generate master cell banks (MCBs) at Henogen/Novasep, Gosselies, Belgium. The MCBs were subsequently used to produce the GMP grade Drug Substances at Fraunhofer IME, Aachen, Germany.

### Upstream processing of PfAMA1-dico proteins

Cultivation was basically as described for PfAMA1 FVO [17], with two major and some minor adaptations. The first major adaptation was a change in the medium, essentially using 20% of the high salt medium that was originally used: 55 kg water, 504 g 85% H_3_PO_4_, 162 g MgSO_4_.7H_2_0, 12.6 g CaSO_4_, 50.4 g KOH, 200 g K_2_SO_4_, 10.5 g EDTA, 375 g NH_4_Cl, 4.06 kg 85% glycerol solution, and 780 g of a modified PTM1 trace salts solution (975 g water, 9.2 g H_2_SO_4_, 0.6 g CuSO_4_.5H_2_0, 0.08 g NaI, 3.0 g MnSO_4_.H_2_0, 0.20 g Na_2_MoO_4_.2H_2_O, 0.02 g H_3_BO_3_, 20 g CoCl_2_.6H_2_0, 0.02 g ZnCl_2_, 65 g FeSO_4_ .7H_2_0 and 0.2 g biotin per litre)). The second major adaptation was the use of an Alcosens online methanol probe and Acetomat controller (Heinrich Frings GmbH, Bonn, Germany) enabling control over the methanol concentration during the induction phase of the fermentation. Moreover, a Foamex 25G mechanic foam-breaking device (Heinrich Frings) was used, rendering (additional) addition of antifoam during fermentation unnecessary.

Briefly, 1 mL of a freshly thawed MCB vial was transferred to 400 mL of YSG^+^ medium (6 g Yeast extract, 5 g soy peptone, 23.5 g 85% Glycerol, 980 g water, pH 6.0) and subsequently cultivated under aerobic conditions at 30°C for 10–14 hours.

300 mL of the preculture was transferred to a 20-L in-situ sterilized bioreactor (Applikon, Schiedam, The Netherlands) containing 10 L YSG^+^ medium fortified with 2 g Struktol J673 antifoam. Cultivation was carried out at 30°C, 0.5 vvm 300 rpm, 0.2 barg headroom pressure without pH control for 10–14 h until an OD_600_ of 8–16 was reached. The content of the 20 L fermenter was transferred to a 140 L fermenter filled with approximately 63 L of the modified mineral medium. Cultivation was carried out as above, with pH set at 6.0. Approximately 17 h after inoculation, when a rise in the dissolved oxygen concentration and pH indicated depletion of the carbon source, 4.5 kg of a 45% (w/v) glycerol solution was fed to the medium at a fixed rate of 14 ml/min. As soon as the entire glycerol feed was consumed and when the in-process control acceptance criterion of OD_600_>220 was met, the headroom pressure was decreased to 0.2 barg again and a pulse of 75–175 mL of methanol was added. When this pulse was used up, again indicated by a rise in dO_2_, one or two more pulses of similar volume were added. After the last pulse, on-line methanol control was started and a concentration of 0.06–0.1% of methanol was maintained constant for 12 to 16 hours.

### Cell separation

Prior to harvesting, the fermenter medium was cooled to a temperature below 10°C while the pH was raised to 7.0 by addition of 25% ammonia. The bulk cell mass was removed by centrifugation in a CARR P6 semi-continuous centrifuge. The centrifuge centrate was passed sequentially over a 1.5 μm-rated depth filter (Sartoclear F4HP 10" or 20" Maxicaps, Sartorius Stedim Biotech, Göttingen, Germany) followed by filtration across 0.45/0.2 μm membrane filters (Sartopore 2 H9 or H0 Midicaps, Sartorius).

### Downstream processing of PfAMA1-DiCos

#### Immobilized Metal Affinity Chromatography

PfAMA1-DiCo proteins were captured from the fermentation supernatant by immobilized metal affinity chromatography (IMAC), essentially as described before [17]. The PfAMA1-DiCo 25–545 proteins inherently bind to immobilized Cu^2+^ although the constructs do not contain a hexahistidine-tag, likely due to the high number of histidines in the prodomain. Briefly, 440 mL of Chelating Sepharose Fast Flow matrix (GE Healthcare, Freiburg, Germany) was packed into an Axichrom 70 column (GE Healthcare) and activated with 25 g/L CuSO_4_. The column was equilibrated with buffer (50 mM NaP_i_, 200 mM NaCl, 10 mM imidazole, pH 7.1 +/-0.1, using an ÅKTA^™^ Pilot chromatography controller (GE Healthcare). Fermentation supernatant was passed over the charged column at a rate of 150 ml/min or 4 cm.h^-1^. Fresh Chelating Sepharose matrix was used for each PfAMA1-DiCo.

After loading, the column was washed using equilibration buffer until the UV signal was below 45 mAU. Protein was eluted using 50 mM NaP_i_, 200 mM NaCl, 125 mM imidazole, pH 7.4 +/- 0.1. The UV signal (280 nm) was used to automatically collect the elution peak into a disposable Flexboy bag of appropriate size (Sartorius, Göttingen, Germany) starting when the UV signal exceeded 100 mAu and ending when the signal fell below 200 mAu. The IMAC eluates were stored at 2–8°C until further processing. Stability of the proteins in the eluates for up to 7 days at 2–8°C was verified before (data not shown).

### Hydrophobic Interaction Chromatography (HIC)

An Axichrom 50 column (GE Healthcare) was packed with approximately 250 mL Butyl 650M Toyopearl (Tosoh Bioscience, Stuttgart, Germany) using the same ÅKTA^™^ Pilot chromatography controller as before. The matrix was washed with equilibration buffer (10 mM NaP_i_, 161 g/L ammonium sulphate (AMS) (30% saturation), adjusted with 10 M NaOH to pH 7.4). After loading the IMAC eluate, that first was fortified with a 80% saturated AMS solution to reach 30% saturation, the column was washed with equilibration buffer until the Absorbance at 280 nm was below 50 mAu. Proteins were eluted with equilibration buffer without AMS. The elution peak was fractionated automatically between 150 (rising) and 450 (falling) mAu. The eluates were stored at 2–8°C overnight.

### Filtration

As a third purification step, essentially to remove a major impurity at 20 kDa, a 30 kDa diafiltration/ultrafiltration step was employed. One Sartocon 30 kDa Hydrosart Tangential-Flow-Filtration (TFF) cassette with a 0.02 m^2^ filtration area (Sartorius Stedim Biotech, Göttingen, Germany) was used in a Sartoflow alpha TFF device (Sartorius), prepared according to the manufacturer's instructions, using diafiltration buffer (10 mM NaP_i_, 500 mM NaCl, pH 7.4 +/- 0.1. HIC-eluates were diluted 1:5 using the same buffer. Diafiltration was carried out at applying approximately 1.9 bar pressure across the membranes. A constant retentate volume was maintained until at least 7.5 L of permeate was generated. At this point, the TFF device was employed in the ultrafiltration mode and the retentate was concentrated to approximately the weight of the HIC-eluate before dilution. The retentate was immediately subjected to the final size exclusion/buffer exchange step.

### Buffer exchange

A BPG 100 column (GE Healthcare) was packed with approximately 1060 mL exclusion chromatography matrix (Sephadex G25; GE Healthcare) and equilibrated using 0.37 g/L NaH_2_PO_4_·2H_2_O, 0.45 g/L K_2_HPO_4_ and 0.0466 g/L EDTA adjusted to pH 6.8 with HCl, using the Åkta Pilot chromatography controller, as before. The diafiltration/ultrafiltration retentate was loaded and eluted using the equilibration buffer. The elution peak was automatically collected between 10 (rising) and 25 (falling) mAu at 280 nm. The purified Drug Substances were stored in Flexboy bags at -20°C.

### Drug substances release testing

The following assays were applied as Drug Substances release tests:

#### Identity by SDS-PAGE and western blotting

SDS-PAGE was carried out using NuPAGE Novex 10% Bis/Tris pre-cast gels (Life Technologies GmbH, Darmstadt, Germany) in MOPS buffer according to manufacturer's instructions. Samples were loaded under reducing and non-reducing conditions and stained with Coomassie-staining procedures (Safe stain, Life Technologies) or silver-staining methods [18]. For western blotting, samples were loaded under non-reducing conditions, blotted to nitrocellulose membranes and PfAMA1-DiCos were detected using the reduction-sensitive anti-PfAMA1 mAb 4G2 [19] and Goat-anti-rat-AP-labelled pAb (Dianova, Hamburg, Germany), using NBT/BCIP as detection reagents.

#### Identity/Purity by Analytical Size-Exclusion High Performance Liquid Chromatography (SEC-HPLC)

SEC-HPLC was performed using PBS, pH 7.4 as mobile phase on an Åkta Purifier Basic (GE Healthcare) and a pre-packed Superdex 200 10/300 GL column (GE Healthcare). The flowrate was 0.5 ml.min^-1^ and detection was at 220 nm.

#### Protein content

Protein content was determined by μBCA, using a commercially available kit (Pierce, Rockford, IL, USA) according to the instructions of the manufacturer.

#### PfAMA1-DiCo content

Calibration-free concentration assay (CFCA) determination of the Drug Substance was carried out using a BiaCoreT200 device according to the manufacturer's manual. Briefly, 10230 RU of mAb 4G2 [19] was immobilized on a CM-5 surface chip. PfAMA1-DiCo Drug substance was passed over this column in HBS-EP buffer (10 mM HEPES, 150 mM NaCl, 3 mM EDTA, 0.05% Polysorbate 20, pH 7.4) at 25°C. Flow rates of 3, 6, 12, 50, 75 and 100 μL/min were used, contact time was 90 s. The surface was regenerated using citrate buffer (500 mM Na_3_Citrate adjusted to pH 3.0 with phosphoric acid) with a contact time of 60 s. The Biacore T200 evaluation software was used to calculate the concentration of PfAMA1 with binding activity for mAb 4G2. The diffusion coefficient of the PfAMA1 analytes was calculated from their molecular mass with the equation and assuming a globular protein with frictional ratio of 1.2 and a buffer viscosity relative to water of 1.

#### Residual host cell protein (HCP) content

A quantitative anti-*Pichia pastoris* HCP ELISA kit, commercially available from Cygnus Technologies, Southport, NC, could not be used due to cross-reactivity with the PfAMA1-DiCo products. Hence, residual host cell protein determination was carried out using a combination of SDS-PAGE/silver staining and western blot methods. Confirmation was obtained using MS analysis.

#### Residual DNA content

Residual DNA content was determined using the Threshold-system (Molecular Devices Corporation, Sunnyvale USA), subcontracted to Newlab AG, Erkrath, Germany.

#### Endotoxin determination

Endotoxin content was measured using the Endosafe PTS reader and chromogenic LAL determination kits according to manufacturer's instructions (Charles River, Wilmington, MA, USA).

#### Other residuals

Analysis of residual copper (ICP-OES/MS), methanol (GC) and imidazole (HPLC), all putative process-related impurities, was outsourced to CBA, Kirkel, Germany.

#### Total viable count (‘bioburden’)

The m*e*thod used complies with the Pharmacopea Europea 2.6.12 “microbial enumeration”membrane filtration test. Incubation was on R2A and TSA media at 30°C and 25°C, respectively, for 3–5 days [20].

### Drug substances characterization tests

The following additional Characterization tests were applied on the Drug Substances:

#### Identification of impurities in the Drug Substances using ESI-TOF Mass Spectrometry

Eluated fractions of individual DiCo1, DiCo2 and DiCo3 HIC runs were separated on SDS Gels. Bands visible after coomassie staining were cut out, trypsin-digested (standard protocol) and the peptides purified using a nano-HPLC LC-Packing (Dionex/Thermo Fisher Scientific, Dreieich, Germany) and injected with the Advion Triversa nanomate (Advion Ithaca, NY, US) in an ESI QUAD TOF QTOF 2 Mass spectrometer (Micromass, Waters S.A.S. Saint-Quentin, France). The peptides found were subjected to a homology search (Mascot, www.matrixscience.com, NCBI database) to identify the proteins as product, product-related or process-related impurities.

#### N-terminal sequencing

N-terminal protein sequencing was carried out as a characterization test using Edman degradation [21], and was subcontracted to Toplab, Martinsried, Germany.

#### Conformational integrity

As a surrogate measure of conformational integrity, the PfAMA1-DiCo Drug Substances were incubated with the reduction-sensitive mAb 4G2 [19] in a 2:3 or 2:5 excess (on a stoichiometric basis) and subsequent analysed by analytical SEC, using PBS, pH 7.4 as the eluent. Detection was with an UV-detector at 220 nm.

#### Mass spectrometric analysis of PfAMA1-DiCo proteins

Size determination of the individual PfAMA1 DiCo protein purified bulk API, was outsourced to the University of Hasselt, Belgium, who carried out MALDI-TOF-Mass spectroscopy, using a Bruker-Daltonics Ultraflex III" MALDI-TOF/TOF.

### Formulation, filling and lyophilisation of the drug product

Process development for formulation, filling and lyophilisation was performed at Nova Laboratories, Leicestershire, UK. Purified bulk material of individual PfAMA1-DiCos (DiCo1, 5.08 mg/mL: DiCo2, 4.96 mg/mL: DiCo3, 4.13 mg/ml) were mixed in a 1:1:1 stoichiometric ratio (0.327:0.335:0.403 g). Aliquots of 0.213 g of the resulting mixed PfAMA1-DiCo solution were diluted to 200 μg/mL using five different buffer formulations: 1) 5% w/v D-mannitol, 5 mM NaH_2_PO_4_/K_2_HPO_4_, 0.125 mM EDTA, pH 6.8; 2) 5% w/v sucrose, 5 mM NaH_2_PO_4_/K_2_HPO_4_, 0.125 mM EDTA, pH 6.8; 3) 10% w/v trehalose dihydrate, 5 mM NaH_2_PO_4_/K_2_HPO_4_, 0.125 mM EDTA, pH 6.8; 4) 9% w/v trehalose dihydrate/1%w/v D-mannitol, 5 mM NaHPO_4_/ K_2_HPO_4_, 0.125 mM EDTA, pH 6.8; and 5) 5% w/v sucrose, 0.125 mM EDTA, 25 mM L-histidine, pH 6.8. Formulation 2 was orinially used for excipitation of the PfAMA1 FVO Drug Substance [17].

Each formulated PfAMA1-DiCo solution was single filtered and volumes containing 60 μg PfAMA1-DiCo Drug Substances were dispensed into 2 mL neutral type I clear glass injection vials (Schott Glaskontor article code: 04110261), and a 13 mm ‘FluroTec’ coated lyophilization stopper (West article code: V2-F8, D713, B2-40) was positioned in the neck of each vial.

All vials were immediately lyophilized in a VirTis Genesis SQ12EL lyophilizer starting with a thermal treatment, followed by 2 drying phases. Thermal treatment started with 15 min at 15°C. Then cooling to -45°C was started (240 min, ramp), followed by 180 min at -45°C. First drying cycle was started by putting on the vacuum at 200 mTorr, keeping the vials at -45°C for 150 min, followed by ramping to -30°C in 240 min, and subsequently leaving the vials at -30°C for 1800 min, all at 200 mTorr. During the secondary drying phase, temperature and vacuum were ramped to 20°C and 50 mTorr respectively, in 480 minutes. Subsequently, these conditions were maintained for 180 min, while in the post-drying step these conditions were continued for another 360 min.

The Drug Product was lyophilized using excipient 2 with a slightly adapted lyophilisation cycle: Thermal treatment started with 60 min at 5°C, followed by cooling to -45°C (200 min, ramp). This temperature was maintained for 60 min. Subsequently the temperature was ramped up to -2°C (in 450 minutes), and maintained for 480 min. Temperature was brought back to -45°C again (ramp, 180 min) and maintained for one more hour. First drying cycle was started by putting on the vacuum at 100 mTorr, keeping the vials at -45°C for 180 min, followed by ramping to -33°C in 120 min, and subsequently leave the vials at -33 for 1320 min, all at 100 mTorr. During the secondary drying phase, temperature and vacuum were ramped to 20°C and 50 mTorr respectively, in 480 minutes. Subsequently, these conditions were maintained for 300 min, while in the post-drying step these conditions were continued for another 240 min.

### Drug product release testing assays

Dissolution rate, appearance (before and after dissolution), pH and sterility [20] were determined using standard protocols. Identity was assessed by reducing and non-reducing SDS-PAGE with silver staining and non-reducing western blot using mAb 4G2 for detection and by SEC-HPLC, as described above for the Drug Substance. Purity was determined simultaneously by SEC-HPLC. Protein content was determined by μBCA, Endotoxin by LAL assay, similar to the Drug Substance release assays (see above).

#### Moisture content

Moisture content was determined using the Karl Fischer assay.

#### Sub-visible particle concentration

The sub-visible particle concentration was determined by the light obscuration method, in accordance with Ph.Eur. 2.9.19 / USP <788>. Both assays were performed at Novalabs Laboratories.

#### Individual DiCo content by competition ELISA

The content of individual PfAMA1-DiCos in the final container (stoichiometric amount of PfAMA1-DiCo1, DiCo2 and DiCo3) was determined using a competition ELISA, basically as described by Kusi and co-workers [22].

To determine the individual antigen content in a sample, 96-well micro-titre plates were coated with 1 μg/mL of PfAMA1- DiCo1 (16 h, 4°C). After blocking for 1 h at 37°C with blocking buffer (PBS fortified with 3% (w/v) BSA and 0.05% (v/v) Tween 20), PfAMA1-DiCo1 containing samples (i.e PfAMA1-DiCo Drug Product), standards and anti-PfAMA1-DiCo1 specific IgG were added (alone and in combination) and incubated for 1 hour at RT. The plates were washed (PBS, pH 7.4 fortified with 1% (w/v) BSA and 0.05% Tween 20) and subsequently alkaline phosphatase conjugated goat anti-rabbit IgG (Dianova) was added, again followed by incubation for 1 hour at RT. Plates were washed as before and developed in DEA buffer (0.15% MgCl_2_.6H_2_O, 0.01% diethanolamine, pH 9.8) containing PNPP substrate (1 mg/mL) and read at 405 nm. In similar fashion, PfAMA1-DiCo2 and DiCo3 individual antigen content were determined. To generate the PfAMA1-DiCo-specific IgG, eg PfAMA1-DiCo1, pooled sera of PfAMA1-DiCo1-immunized rabbits were consecutively passed over columns with immobilized PfAMA1-DiCo2 and immobilized PfAMA1-DiCo3 until binding to PfAMA1-DiCo2 and PfAMA1-DiCo3 was less than 5% than binding to PfAMA1-DiCo1.

Buffer exchange to PBS, pH 7.4 was achieved by concentration/dilution in ultrafiltration units (Millipore) with a cut-off of 100 kDa. Specific sera for PfAMA1-DiCo2 and DiCo3 were prepared likewise.

#### Abnormal toxicity

Five healthy mice and two healthy guinea pigs were injected intraperitoneally with one human dose of the vaccine and observed for 7 days. The test is passed if none of the animals shows signs of ill health, according to the European Pharmacopoeia 2.6.9 [20]. This work was carried out by Confarma France S.A.S, Hombourg, France, under a general ethical approval for their test facility (D-68-144-03), listing assay types and species. This approval was given after external audit of the facilities to ensure compliance with the legislation regarding housing and treatment of the test animals at the time of the experiment.

Review of critical phases was performed by the Ethics Committee of Confarma France.

### Immuno-potency assay development

Female BALB/c mice were immunised subcutaneously with PfAMA-1 DiCo 1, DiCo2 and DiCo3 in a 1:1:1 ratio mix. Five antigen dosages (0.01, 0.03, 0.1, 0.3 and 1 μg) were mixed to either GLA-SE (5 μg of Glucopyranosyl Lipid Adjuvant and 2.0% of Stable Emulsion (Infectious Diseases Research Institute, Seattle, USA)) or Rehydragel (0.2% of aluminium oxide (General Chemical, USA)). Mice were immunised at day 0 and 28 by subcutaneous injection of 100 μL of the reconstituted vaccine. Blood samples were collected at day 0 and 42, diluted sera (1:120) were analysed by ELISA (detecting PfAMA1 DiCo1, DiCo2 or DiCo3 antibodies).

The dose for the potency release assay was selected on basis of the results obtained. See below.

This work was carried out by Confarma France S.A.S, Hombourg, France, under a general ethical approval for their test facility (D-68-144-03), listing assay types and species. This approval was given after external audit of the facilities to ensure compliance with the legislation regarding housing and treatment of the test animals at the time of the experiment.

Review of critical phases was performed by the Ethics Committee of Confarma France.

### Immuno-potency release assay

One μg PfAMA1-DiCo Drug Product, formulated with either Alhydrogel or GLA-SE was injected subcutaneously in 11 mice at day 0 and at day 28. Additionally, 0.1 μg was used in a similar fashion. 0.1 μg is assumed to be more sensitive to changes in the Drug Product and will be used as an early warning system for loss of immuno-potency. Antibody titers against PfAMA1 DiCo1, PfAMA1 DiCo2 and PfAMA1 DiCo3 were determined and the titre increase was calculated by dividing the Day = 42 titre by the Day = 0 titre. The acceptance criterion for the release was defined as follows: Seroconversion (defined by the increase in average AMA1-DiCo-specific IgG concentration) should be more than four-fold higher on day 42 compared to day 0 in at least 80% of the animals, for the 1 μg dose.

This work was carried out by Confarma France S.A.S, Hombourg, France, under a general ethical approval for their test facility (D-68-144-03), listing assay types and species. This approval was given after external audit of the facilities to ensure compliance with the legislation regarding housing and treatment of the test animals at the time of the experiment.

Review of critical phases was performed by the Ethics Committee of Confarma France.

### Drug product characterization test

Conformational integrity after mixing, lyophilisation and reconstitution was determined for the Drug Product with mAb individual, followed by SE-HPLC, essentially as described for the Drug Substances (see above).

As a second surrogate marker for conformational integrity, the level of free cysteines of the Drug Product was determined using the “Free Sulfhydryl Quantitation Kit” (Life Technologies), according to the instruction of the manufacturer. Bovine Serum Albumin was used as a control.

### Pharmacotoxicity study

The pharmaco-toxicity study setup was in accordance with the European Medicines Agency /Committee for Proprietary Medicinal Products (EMA/CPMP): Note for guidance on preclinical pharmacological and toxicological testing of vaccines (http://www.ema.europa.eu/docs/en_GB/document_library/Scientific_guideline/2009/09/WC500003809.pdf) and the Guideline on adjuvants in vaccines for human use (http://www.who.int/biologicals/publications/trs/areas/vaccines/nonclinical_evaluation/en).

This work was carried out by WIL Research B.V., ‘s-Hertogenbosch, The Netherlands. The protocol was reviewed and agreed by the Animal Welfare Officer and the Ethical Committee of WIL Research Europe B.V. (DEC 10–13) as required by the Dutch Act on Animal Experimentation (February 1997).

Briefly, Albino New Zealand White Rabbits (8 males and 8 females (12–14 weeks old, 2–3 kg at start of study)) were injected intramuscularly with 0.5 mL PfAMA1-DiCo Drug Product (DP) (full human dose: 50 μg) on days 1, 15, 29 and 43 alternatingly in the hind limb. 5 animals per sex of each group were sacrificed on day 45, the remaining animals on day 65 (recovery). Treatment groups were: PfAMA1 DiCo DP + Saline; PfAMA1 DiCo DP + GLA-SE (5 μg GLA in 2% oil); GLA-SE; Saline; PfAMA1 DiCo DP + Alhydrogel (0.85 mg aluminium (Al^3+^)).

Animals were scored for mortality (at least twice daily), clinical signs (daily), skin irritation (24 and 48 hours after each administration), body weight (weekly), food consumption (twice weekly), ophthalmoscopic examination (during pre-test and at the end of treatment and recovery period), clinical pathology (during pre-test and on days 3, 17, 31, 45 and/or 65), macroscopy and organ weights at termination and histopathology on a selection of tissues. Blood samples for immunogenicity were taken pre-dose and at necropsy (either day 45 or day 65).

### Drug product stability

A real-time stability study was designed and carried out on the Drug Product stored at -20°C, according to the ICH Q5A guideline (http://www.ema.europa.eu/docs/en_GB/document_library/Scientific_guideline/2009/09/WC500002801.pdf). At each time point (3, 6, 9, 12,18 and 24 months) a full Drug Product release test was performed on the samples, with the exception of the sub-visible particle counting that is performed with a minimal frequency of once every 6 months, the sterility test that will only repeated at the last sampling time point (month 36). Moreover, the endotoxin level, the abnormal toxicity and the potency tests of the Drug Product formulated with GLA-SE were not evaluated during the real time stability study.

During the stability study, the Drug Product by assessed by Surface Plasmon Resonance (see determination of Total DiCo Content) and by reversed-phase HPLC, as follows: DiCos 1–3 were separated on a Jupiter C18, 250 x 4.6 mm x 3 μm column (Phenomenex, Aschaffenburg, Germany) at 40°C using 10% acetonitrile with 0.1% trifluoroacetic acid (TFA) as eluent A and 80% acetonitrile with 0.1% TFA as eluent B. After equilibration with 14% of eluent B, 100 μl of the reconstituted drug product was injected. DiCos 1–3 eluted sequentially in a linear gradient at approximately 36.5 to 39% eluent B. Flow rate was 0.5 mL/min and detection was at 210 nm.

### Additional stability tests

Real time stability of the Drug Product was also assessed after storage at 4–8°C, with the same time intervals and the same assays as for the genuine stability study.

A stability stress test was also carried out on the Drug Product. Final containers were stored for 7 days at 30°C. Again, a full Drug Product release test was performed, with the exception of the endotoxin level, sterility, abnormal toxicity and potency.

Finally, short-term stability of the Drug Product in the presence of adjuvants was assessed. To this end the Drug Product was formulated with either Alhydrogel or GLA-SE and stored in glass vials at 5°C or at 25°C for 24 hours and tested for biological activity in potency assays. Additionally, the formulae were broken [13] and the fractions containing the proteins were analysed by methods described for the Drug Product release: pH, reduced and non-reduced SDS-PAGE, western blot, protein content, PfAMA1-DiCo individual content.

ELISA and SDS-PAGE were also used to estimate the adsorption of the Drug Product to Alhydrogel.

To investigate the compatibility and short-term stability of the Drug Product formulated with GLA-SE, particle size and zeta potential measurements were performed after 10 min, 1 hour, 24 hours and 7 days storage at 2–8°C, 15–25°C and 37°C. This work was carried at the Vaccine Formulation Laboratory in Lausanne, Switzerland.

## Results

### Construction of PfAMA1-DiCo producing Pichia clones

The construction of Pichia clones was done according to published methods. The sequences used here differ from the published sequences [15] by the presence of the PfAMA1 FVO prodomain (aa 25–96) and a mutation in the conserved domain II loop (K376R), as preliminary evidence suggested that this would abrogate the susceptibility to proteolytic attack.

### Process development

For the first generation PfAMA1 GMP production, Faber *et al*. reported a specific and partial proteolytic cleavage of the secreted product, which became evident only in SDS-PAGE under reducing conditions [17]. Several studies show that optimizing the process parameters (eg T, t, feed rate) and adaptation of induction strategies (eg use of mixed feeds, use of low levels of methanol or methanol limitation during the induction phase) during the fermentation may improve both quality and quantity of the target products [23–30], while the use of a constitutive GAP promotor before the *pfama1* gene in *Pichia pastoris* did not improve yield or quality of the PfAMA1 protein produced [B.W. Faber, unpublished results].

Consequently, the upstream process development preceding the GMP production of the PfAMA1-DiCos described below aimed at improving the stability, while not substantially lowering the yield.

### Production and purification of PfAMA1-DiCos using *Pichia pastoris*

About 40 non-GMP development runs in the 4- and 20-L scale as well as technical runs in the final 70-L scale were carried out prior to the GMP campaigns. The original basal salts composition was drastically reduced to approximately 1/5^th^ in most salts, as described before [31–33], thus reducing the osmotic stress during high-cell-density fermentation but still supplying all necessary elements to promote growth to cell densities well above 200 g wet weight per L. Because of hints that copper and cobalt ions could increase proteolytic degradation of secreted PfAMA1-DiCos (B.W. Faber, unpublished observations), the concentration of Cu in the trace salts solution was reduced to 10% (as in [17]) and of Co reduced to 40% of the concentration in the standard PTM1 trace salts formulation. These modifications are in accordance with recent awareness that while *Pichia* readily grows to very high cell densities, the standard conditions were developed with single-cell-protein-production in mind. This may exert stress on the cells, which in turn may be suboptimal for heterologous protein production [34, 35]. Fermentations at more neutral or slightly basic pH did not improve quality or yield of the proteins (data not shown). Lower temperature during induction, as suggested by temperature-limited fed-batch cultivations, was not tested [24, 36]. In several initial fermentation runs a sudden and dramatic onset of proteolytic degradation of the secreted PfAMA1-DiCo proteins was observed to the extent that the product disappeared completely from the supernatant. Addition of 5 g/L NH_4_Cl to the medium solved this problem. The final cultivation strategy was chosen for simplicity, robustness and predictability and used an identical strategy for all three PfAMA1-DiCo-expressing strains: cells were grown until the initial glycerol was depleted, then a fixed-rate glycerol feed was started until a fixed amount of glycerol was fed. At this point, an in-process control verified that the OD_600_ was above 220. The yeast in the fermenter was adapted to the inducing agent methanol by addition of small amounts of methanol in a pulsed manner. dO_2_ and pH signals were used to detect depletion, after which the next addition was done. This adaptation process lasted for 4–6 hours. Subsequently, the methanol concentration was maintained at approximately 0.07% v/v for 12 to 16 hours using online methanol analysis with a dose monitor. In the GMP-campaign, the three fermentations were run in three subsequent weeks.

**Fig 1:**
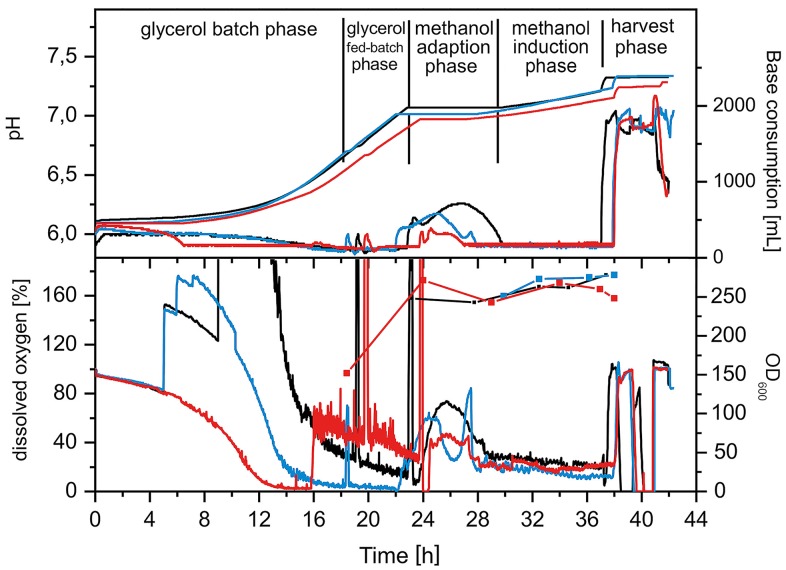
Fermentation profiles for the GMP runs for PfAMA1-DiCo Drug Substances. Upper panel. Base consumption and pH. Base consumption (NH_4_OH addition) is related to growth and metabolic activity, since NH_4_^+^ is the single source of nitrogen. Addition of base compensates the acidification during aerobic growth of microbes in mineral media. A pH setpoint of pH = 6.0 was used throughout the fermentations. Lower panel. Biomass and dissolved oxygen. The offline determined OD_600_ was taken as a measure for biomass (1 OD_600_ ~ 1 g/L w/w). The curves show the typical batch, glycerol fed-batch, methanol adaptation and methanol induction phases of Mut^s^ Pichia strain fermentations. Black lines; PfAMA1 DiCo1; blue lines, PfAMA1 DiCo2; red lines, PfAMA1 DiCo3.

Fig 1 shows key on-line and off-line parameters for the three fermentations.

Cell separation was carried out using a semi-continuous solids discharging centrifuge. The centrate or supernatant was filtered using a depth-filter and a membrane filter in sequence to obtain a cell-free supernatant. Downstream from the centrifugation step, only disposable tubing, filters and liquid containers were chosen to minimize cleaning activities, minimize the risk of cross-contamination and to allow for the rapid succession of the three batches.

The clarified supernatant was first subjected to immobilized metal affinity chromatography (IMAC) using copper as the affinity ligand. During process development, zinc and nickel ions had been tested as alternatives. The binding/elution characteristics were found most suitable using copper (data not shown). Intrinsic properties (i.e the presence of the PfAMA1 FVO prodomain) of the PfAMA1 constructs allow for the use of IMAC as a capture step, although the constructs do not contain a hexa-histidine tag [B.W. Faber, unpublished observation]. From approximately 60–70 L of clarified fermentation supernatant of the three batches, 350, 322 and 316 g main eluate fractions containing PfAMA1 DiCo proteins 1, 2 and 3, respectively, were obtained.

The eluates were adjusted to 30% saturation of ammonium sulphate and passed over a hydrophobic interaction chromatography (HIC) column. For the three PfAMA1-DiCo proteins, 151, 153 and 154 g main eluates were obtained in low-salt buffer. The HIC step served to remove the yellowish to greenish pigmentation that has been reported frequently in IMAC eluates of methanol-fed *Pichia* fermentation supernatants [37–40]. At this point, an additional 30-kDa diafiltration/ultrafiltration step was introduced, compared to the production process of the first generation PfAMA1 [17]. The reason was a 20-kDa product-related impurity that was observed during the process scale-up. This step was shown to quantitatively remove the 20-kDa impurity (data not shown).

Finally, a buffer exchange step was performed through size exclusion chromatography. For the three PfAMA1-DiCo proteins, 346, 307 and 324 g of purified bulk material of 5.1, 5.0 and 4.1 mg/mL total protein content were obtained, translating to a batch yield of approximately 1.5 g per 70 L fermentation. The purified bulks were filled into single-use bags and stored at -20°C. Table 1 shows the efficiency of the different steps of the purification.

**Table 1:** Purification table for PfAMA1-DiCo1, PfAMA1-DiCo2 and PfAMA1-DiCo3 proteins during the actual GMP production.

Step	Total weight (g)	Concentration^a^(mg·mL^-1^)	Purity^b^ (%)	DiCo content^c^ (g)	Step efficiency(%)
Fermenter supernatant	approx. 60.000	n.d.	n.d.	approx. 6^d^	100
IMAC eluate	350/322/316	7.3/7.6/6.9^f^	91/90/86 ^f^	2.3/2.2/1.9^f^	approx. 35
HIC eluate	150	14.0/13.3/11.7^f^	90/91/88 ^f^	1.9/1.8/1.6^f^	82–84
Dia/Ultrafiltration Retentate	150	10.3/10.4/9.3^f^	96/n.d./97 ^f^	n.d.	n.d.
GF eluate	346/307/324	5.1/5.0/4.1^f^	97/98/98 ^f^	1.7/1.5/1.3^f^	81–89^e^
Overall					23–26^g^

^a^ Concentration measurements were carried out using protein absorption at 280 nm

^b^ Determination of purity was done by analytical size-exclusion chromatography.

^c^ DiCo content was calculated from weight, concentration and purity determination

^d^ Estimated from Coomassie-stained SDS-PAGE band intensity

^e^ Calculated as combined efficiency for DF/UF and GF process steps

^f^ For PfAMA1 DiCo1, DiCo2 and DiCo3, respectively

^g^ Calculated as combined efficiency of all steps

### Drug substances release testing

**Fig 2:**
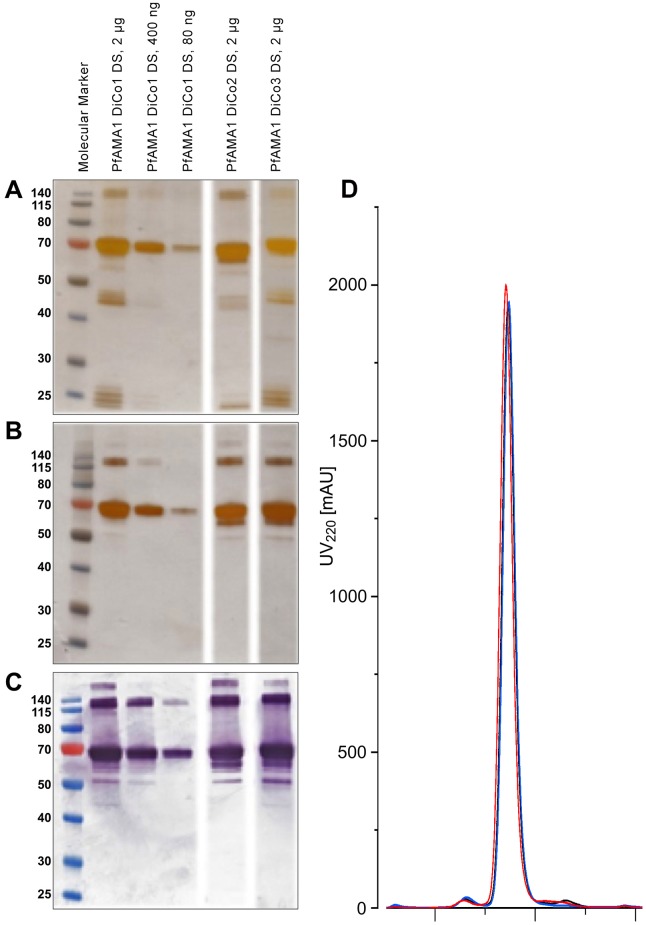
SDS-PAGE, Western Blotting and SEC profiles of the Drug substances PfAMA1 DiCo1, PfAMA1 DiCo2 and PfAMA1 DiCo3. Panel A: Reducing SDS-PAGE, silver-stained. Panel B: Non-reducing SDS-PAGE, silver-stained. Panel C: Non-reducing SDS-PAGE and Western-Blot, immune-staining using the reduction-sensitive monoclonal anti-PfAMA1 antibody 4G2. Panel D: SE-Chromatography of each of the PfAMA1 Drug Substances. PfAMA1 DiCo1 (black), PfAMA1 DiCo2 (blue) and PfAMA1 DiCo3 (red) was carried out on a Superdex200 10/300 GL prepacked column (GE Healthcare), using an Åkta Purifier Basic (GE Healthcare). PBS (140 mM NaCl, 10 mM sodium phosphate, 3 mM KCl, pH 7.4, Calbiochem) was used as running buffer at 0.5 mL.min^-1^.

The PfAMA1-DiCo Drug Substances (purified bulks) were analysed using a variety of methods. The purity of the proteins was assessed by reduced and non-reduced SDS-PAGE, western blotting and size exclusion chromatography (SEC) (Fig 2).

The SDS-PAGE analysis shows that the proteins have an apparent molecular weight between 65 and 70 kDa. SEC essentially shows that the purity of the individual PfAMA1-DiCo Drug Substances was over 95% (96.8, 97.6 and 97.7%, respectively), while product related residuals were <3.2% (1.6% dimers; 1.6% fragments; 0.2% aggregates), <2.7% (2.0% dimers; 0.3% fragments; 0.4% aggregates) and <2.4% (1.2% dimers; 1.1% fragments; 0.05% aggregates), for PfAMA1-DiCo1, DiCo2 and DiCo3 proteins, respectively. Moreover, identity was confirmed by western blotting showing binding of the conformational (reduction sensitive) monoclonal antibody 4G2 (Fig 2C). Total viable count of the PfAMA1-DiCo1 and DiCo2 Drug Substance preparations indicated absence of microbial contamination, while the PfAMA1-DiCo DiCo3 Drug Substance had a count of 250 CFU/mL, below the acceptable level of 1000 CFU/mL. The levels of residual DNA (<310 pg/mL for all PfAMA1-DiCo proteins), endotoxin (0.072 EU/mL, <0.05 EU/mL and <0.05 EU/mL for PfAMA1-DiCo1, DiCo2 and DiCo3, respectively) and the levels of the potential process-related residuals MeOH (<10 ppm for all DiCo proteins), Cu^2+^ (580, 385 and 346 ppb for PfAMA1-DiCo1, DiCo2 and DiCo3, respectively) and imidazole (<0.25 μg/mL for all PfAMA1-DiCo proteins) were all below the pre-set criteria.

As the commercial available kit for host-cell-protein determination for Pichia showed cross-reactivity with the PfAMA1-DiCo proteins, a combined approach was taken to determine the levels of residual host proteins, that are normally low for *Pichia pastoris* derived (secreted) therapeutic proteins. Instead, SDS-PAGE with silver-staining and western blotting were used to detect HCPs (S1 Data). These analyses showed that HCP levels of the preparations were well below the acceptable levels of 1%.

### Drug Substance Characterisation Tests

For each PfAMA1-DiCo protein, two N-terminal ends were identified, basically in a one to one ratio. The first, EFQNYW, was the expected N-terminal, the glutamine and phenylalanine encoded by the EcoRI restriction site followed by the beginning of the FVO AMA1 prodomain amino acids, position 25 and further. The other N-terminal that was determined had the sequence SDVYHP, in accordance with a truncated product cleavage behind Lys 34 (AMA1 FVO nomenclature). Mass spectrometry was carried out on PfAMA1-DiCo1, DiCo2, DiCo3 obtained from pre-GMP batches. For each of these proteins two main peaks were observed with MH^+^ values that were close to the theoretical (mono-isotopic) masses of the full-length protein and the full-length protein missing the N-terminal 12 amino acids (DiCo1, observed (full length, truncated)/calculated (full length, truncated): (60732, 59127)/(60773, 59123); DiCo2, observed (full length, truncated)/calculated (full length, truncated): (60903, 59312)/(60758, 59108); DiCo3, observed (full length, truncated)/calculated (full length, truncated): (60489, 58866)/(60622, 58972), confirming the N-terminal sequencing data.

**Fig 3:**
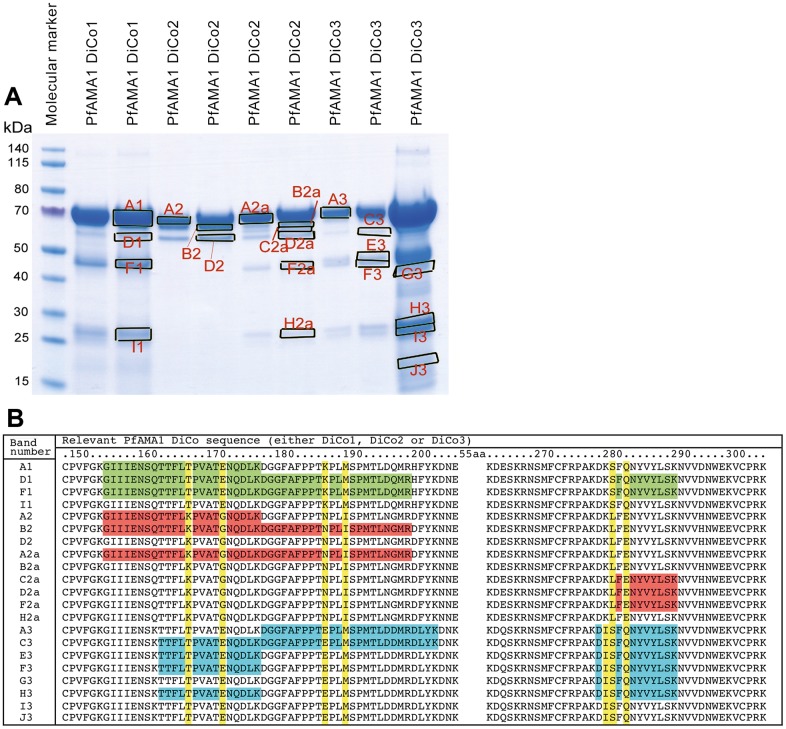
Signature peptide MS analysis of pre-GMP produced PfAMA1 DiCo Drug substance intermediates. Panel A. Overloaded SDS-Gel of HIC eluate fractions with excised bands in marked boxes. Panel B. Examples of signature peptides identified in the 21 excised protein bands for PfAMA1-DiCo preparations. Key to preparations: PfAMA1 DiCo1 (Green), PfAMA1 DiCo2 (Orange) and PfAMA1 DiCo3 (Blue). In yellow the signature amino acids in these peptides, allowing for the differentiation between individual PfAMA1 DiCo proteins. Numbering of the amino acids is as in [15].

An SDS-PAGE with individual purified bulk proteins obtained from preclinical runs (approximately 10–20 μg total protein per slot), was run and stained with Coomassie Brilliant Blue. Individual bands from each lane were isolated (Fig 3A). Subsequent tryptic digestion and isolation of the resulting peptides from each band followed by MS analysis showed that all the identified peptides were derived from the corresponding PfAMA1-DiCos, with only one exception, from a very weak band in PfAMA1-DiCo2.

Moreover, this analysis showed that the mass fingerprints obtained for each PfAMA1-DiCo product are useful for identification, as specific peptides were detected for each of the PfAMA1-DiCo proteins, enabling a qualitative and semi-quantitative analysis of the Drug Product by Mass spectrometry (Fig 3B).

### Exploratory assays for the Drug Substances

#### Conformational integrity of PfAMA1-DiCo Drug Substances

**Fig 4:**
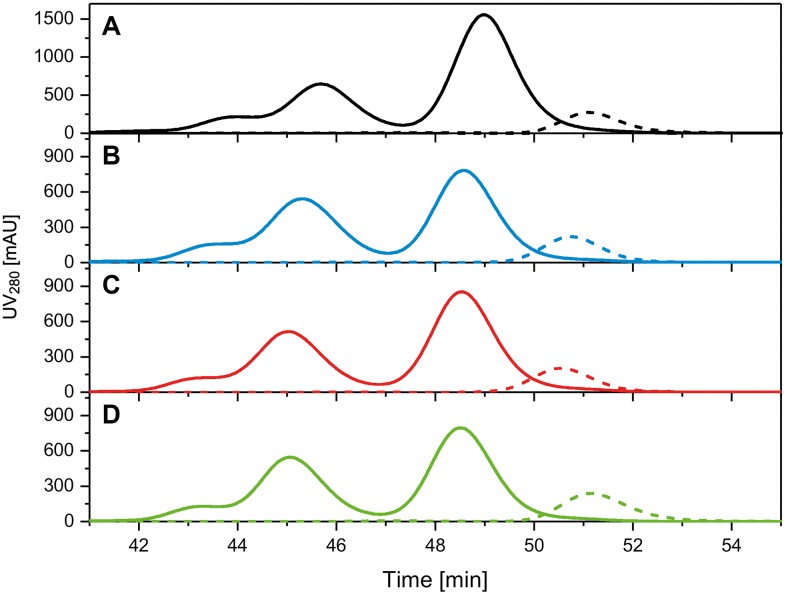
Quantitative binding of Drug Substances and Drug Product to mAb 4G2. Panel A: PfAMA1 DiCo1 Drug Substance (black dotted line) and DiCo1 Drug Substance incubated with a 2.5 fold excess of 4G2 (black solid line). Panel B: PfAMA1 DiCo2 Drug Substance (blue dotted line) and DiCo2 Drug Substance incubated with a 1.5 fold excess of 4G2 (blue solid line). Panel C: PfAMA1 DiCo3 Drug Substance (red dotted line) and DiCo3 Drug Substance incubated with a 1.5 fold excess of 4G2 (red solid line). Panel D. PfAMA1 DiCo1:DiCo2:DiCo3 Drug Substance mixture (1:1:1 w/w/w) (green dotted line) incubated with a 1.5 fold excess of 4G2 (green solid line). PfAMA1 DiCos and mAb 4G2 were mixed and incubated for 1 h at room temperature before injection.

Since it has been shown that immunization with reduced and alkylated AMA1 does not induce functional inhibitory antibodies [41], a size-shift assay was performed to verify correct conformation of the protein. All PfAMA1-DiCo Drug Substances showed quantitative binding with the reduction-sensitive mAb 4G2 (Fig 4).

### Formulation, excipiation and lyophilisation

It was assumed that the formulation that was used for the PfAMA1 FVO product could be improved, as, in the past, a more or less standard recipe with sucrose as cryopreservant was used. This yielded a suboptimal cake, although the stability profile was good [17]. As trehalose or D-mannitol are generally considered to form better cakes than sucrose, these cryopreservants were used in the new formulations. The ‘old’ formulation and four new formulations were used in a preliminary lyophilisation run with preparations produced under GMP, but no longer classified as such. After lyophilisation, the cakes obtained with formulations that contained trehalose or mannitol indeed appeared better that the one obtained with the ‘old’ formulation. See Table 2.

**Table 2:** Analysis of experimental excipients mixed with PfAMA1 DiCo Drug Substances.

		Size Exclusion analytical analysis			
Cryopreservants^a^	Appearance	Main peak (%)	Dimers (%)	Aggregates (%)	By-products (%)
5% D-mannitol	homogeneous white cake	68.51	13.25	16.62	1.61
5% Sucrose	inhomogeneous white cake	94.30	2.64	0.88	2.18
10% Trehalose.2H_2_O	homogeneous white cake	94.82	1.17	3.89	0.13
9% Trehalose.2H_2_O; 1% D-mannitol	nearly homoge- neous white cake	94.57	1.96	2.02	1.45
5% Sucrose; 25 mM L-Histidine	inhomogeneous white cake	81.65	12.06	0.99	2.8

Drug Substances were mixed in a 1:1:1 ratio and subsequently lyophilized in the presence of the excipient, stored for 7–8 days at -20°C and analysed using SE chromatography immediately after dissolving in water.

^a^All lyophilisation buffers also comprised of 5 mM NaH_2_PO_4_/K_2_HPO_4_, 0.125 mM EDTA, pH 6.8

Lyophilized vials of each formulation were stored for 7–8 days at -20°C and the content of the vials was subsequently analysed by SE chromatography. These studies showed that, despite the lesser appearance, the ‘old’ formulation had a very good overall performance with respect to the main peak, levels of dimers, aggregates and by-products (Table 2)> Although both formulations with trehalose also had good performance, albeit that aggregates were higher, it was decided to use the second (old) formulation (5% w/v sucrose, 5 mM NaH_2_PO_4_/K_2_HPO_4_, 0.125 mM EDTA, pH 6.8) for the lyophilisation of the GMP batch, with a slightly adapted thermal treatment and drying cycle (See methods), as the previous vaccine product had been shown to be stable in this formulation [17].

### Drug product release assay

Criteria were pre-set for progression to the phase I clinical trial; *in vitro* analyses required to meet regulatory guidelines included measurement of endotoxin level, appearance before and after reconstitution, dissolution rate, pH (after dissolution in 0.9% NaCl), moisture content, sub-visible particles, sterility, purity, identity, protein content and individual PfAMA1-DiCo content. In addition, animal studies confirmed the immunopotency of the product and absence of abnormal toxicity (Table 3).

**Table 3:** Batch analysis and stability data for PfAMA1-DiCo Drug Product.

Test Parameter	Specification		At release	At T = 0	T = 7 d 30°C	T = 3 m -20°C	T = 6 m -20°C	T = 9 m -20°C	T = 12 m -20°C	T = 18 m -20°C	T = 24 m -20°C
Appearance of lyophilised product^a^	White cake		Pass	Pass	Pass	Pass	Pass	Pass	Pass	Pass	Pass
Appearance of reconstituted solution^a^	Colourless to brownish, clear liquid without visible particles after reconstitution		Pass	Pass	Pass	Pass	Pass	Pass	Pass	Pass	Pass
Moisture content	<4% w/w		<3%	<3%	2.3%	2.0%	2.0%	1.8%	1.8%	1.8%	2.0%
Dissolution rate^a^	<2 minutes		30 sec	30 sec	29 sec	7 sec	17 sec	8 sec	7sec	9 sec	9 sec
Sub-visible particles	≤6000 particles of 10 μm in size or larger per container		25	25	45	n.d.	13	n.d.	32	n.d.	16
	≤600 particles of 25 μm in size or larger per container		2	2	4	n.d.	0	n.d.	3	n.d.	1
Identity western blot	A principle band with relative molecular mass between 65 and 70 kDa		Complies	Complies	Complies	Complies	Complies	Complies	Complies	Complies	Complies
Identity SDS-PAGE	A principle band with relative molecular mass between 65 and 70 kDa		Complies	Complies	Complies	Complies	Complies	Complies	Complies	Complies	Complies
Identity and purity by SE-HPLC (%)	main peak >95%		96.7	97.3	97.5	96.8	97.1	95.8	96.0	95.6	95.4
pH (-)	6.3–7.3		6.5	6.5	6.5	6.4	6.4	6.8	6.4	6.8	6.4
Protein content by μBCA (μg/mL)	79.5–129.5 μg/mL		100.7	103.4	96.1	97.6	91.4	98.4	96.8	106.4	108.1
Total PfAMA1 DiCo content by SPR (μg/mL)	Report results		n.d.	50.4	41.9	44.9	46.7	37.0	39.5	38.8	41.3
DiCo content by competition ELISA (μg/mL)	All three DiCos detectable in the reconstituted mix^b^ Report results^c^	DiCo 1:	Pass	35.1	32.1	50.3	26.4	32.1	45.1	40.8	32.1
		DiCo 2:		34.4	36.5	32.9	35.8	37.0	39.6	30.4	34.4
		DiCo 3:		25.1	35.9	31.5	21.2	30.7	28.3	26.5	28.5
Potency (Alhydrogel)	Not less than 80% seroconversion		Pass	Pass	Pass	Pass	Pass	Pass	Pass	Pass	Pass
Potency (GLA-SE)	Not less than 80% seroconversion		Pass	n.d.	n.d.	n.d.	n.d.	n.d.	n.d.	n.d.	n.d.
Endotoxin	<30 IU/dose (50μg) (eq. to <36 IU/vial)		Pass	n.d.	n.d.	n.d.	n.d.	n.d.	n.d.	n.d.	n.d.
Sterility	Sterile		Sterile	Sterile	n.d.	n.d	n.d.	nd	n.d.	n.d.	n.d.
Abnormal toxicity	No weight loss, no abnormal reaction		Pass	n.d.	n.d.	n.d	n.d.	nd	n.d.	n.d.	n.d.

^a^. Reconstitution with 0.6 ml saline

^b^. Specification at release and for the stress test: 7d at 30°C

^c^. Specification for the real time stability study

n.d.: not done

### Detection and quantification of individual PfAMA1-DiCo proteins in the Drug Product

The release criterion for the Drug Product for the presence of the three DiCo proteins was defined as “Detectable levels of the individual DiCo proteins in the Drug Product using a competition ELISA”. This was done to avoid false disqualification of the Drug Product, as the quantification of the highly similar proteins in the Drug Product proved to be challenging.

Several methodologies were explored to qualitatively and quantitatively analyse the individual PfAMA1-DiCo proteins in the Drug Product (1:1:1 mixture of the three PfAMA1 DiCo proteins). Firstly, the quantitative sandwich-ELISA used for the PfAMA1 FVO vaccine product [17] was tried. Unfortunately, this assay could not be used to quantify the levels of the individual PfAMA1 DiCo proteins in the Drug Product, as it was observed that the affinity of the 4G2 antibody for the different DiCo proteins was too far apart, leading to unreliable results.

Surface Plasmon Resonance was also explored as a methodology to quantify the amount of the individual PfAMA1 DiCo proteins in the Drug Product and to determine the total concentration of the PfAMA1-DiCo Proteins in the Drug Product. This methodology was used in the stability studies to determine the total amount of DiCo protein, however it was not possible to reproducibly quantify the levels of the individual PfAMA1 DiCo proteins in the Drug Product, possibly also related to the different affinities of the PfAMA1 DiCo for the mAb 4G2, and therefore it could not be used as a release assay for individual DiCo content.

Eventually, a quantitative assay was developed based on competition ELISA [22]. This was used as release assay for the quantification of the level of individual PfAMA1 DiCo proteins. Briefly, a series of standards were made with known variable amounts of PfAMA1-DiCo1 and constant amounts of PfAMA1-DiCo2 and PfAMA1-DiCo3. To aliquots of these samples, increasing amounts of specific anti-PfAMA1-DiCo1 antibodies were added and these mixtures were, after a short incubation, added to an ELISA plate coated with PfAMA1-DiCo1 protein. An identical procedure was followed for the Drug Product, dissolved in a known amount of demineralized water. Interpolation of the level of depletion for the Drug Product on those of the known concentrations PfAMA1-DiCo in the mixture enables the quantification of the levels of PfAMA1-DiCo1 in the Drug Product. Similar procedures were followed for PfAMA1-DiCo2 and PfAMA1-DiCo3. At release, the acceptance criteria, defined as mentioned above, were met.

### Stability studies

Real time stability studies were conducted on the Drug Product stored at -20°C. Accelerated studies were performed at 30°C to get insight in the long-term product stability. The competition ELISA described above was used to determine the content of the individual PfAMA1 DiCo proteins during the stability studies (See Table 3). The values obtained (f.e. at T = 0) were 35, 34 and 25 μg/mL of PfAMA1-DiCo1, DiCo2 and DiCo3, respectively, close to the theoretical value of 33 μg/mL for each of the individual PfAMA1 DiCo protein. These results were within the acceptance criteria (Table 3).

**Fig 5:**
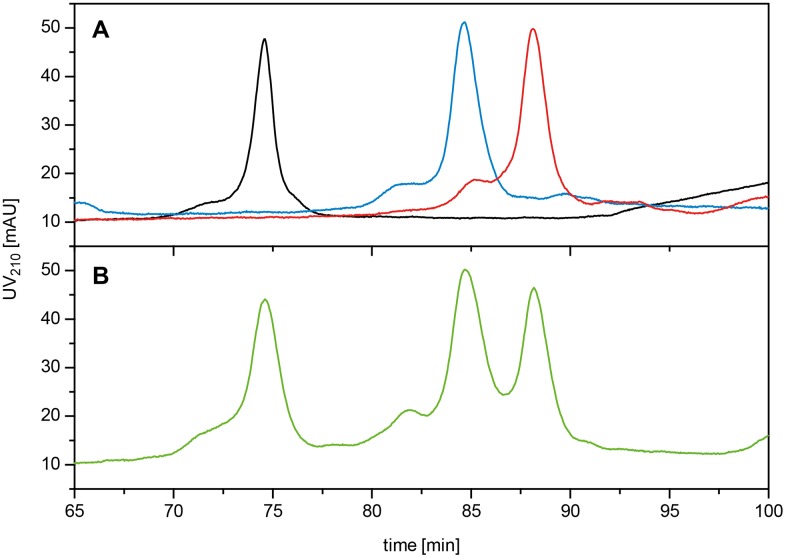
RP-HPLC analysis of individual and mixed PfAMA1-DiCo1, PfAMA1-DiCo2 and PfAMA1-DiCo3 samples. Upper panel: Injection of the individual PfAMA1 DiCo proteins to establish the chromatographic profile of each of these proteins. PfAMA1 DiCo1 in black, PfAMA1 DiCo2, in blue and PfAMA1 DiCo3 in red. Lower panel: Chromatogram of the drug product containing the three PfAMA1 DiCo proteins at equimolar ratios, in green. For chromatographic details see the Methods Section.

As an alternative, explorative assay, a Reversed Phase HPLC method was developed to quantify the individual PfAMA1-DiCo proteins in the Drug Product. This method was only used during the stability study. Fig 5 shows a typical HPLC profile for the individual PfAMA1 DiCo products as well as a 1:1:1 mixture of PfAMA1-DiCo1, DiCo2 and DiCo3, essentially showing that this is a useful alternative assay for the competition ELISA that was used as release assay.

### Immunopotency assay development

It was recognized that the potency assay that was used during the previous clinical trial [17] was not optimal for the detection of loss of potency. A good immune-potency test will not only demonstrate the ability of a vaccine to induce the formation of antibodies in mice (measurement of the sero-conversion rate), as marker for this ability in humans, but will also be able to detect loss in potency over time, and as such, should be designed to be able to do so. The latter aspect is of specific importance for AMA1-based vaccines, as Saul and co-workers have reported that the immune-potency of PfAMA1 formulated with Montanide ISA720 diminishes upon storage [42].

To establish such potency assay, five antigen dosages (0.01, 0.03, 0.1, 0.3 and 1μg) were formulated with GLA-SE or Alhydrogel. BALB/c mice were immunised at day 0 and 28 by subcutaneous injection and day 0 and 42 sera were analysed by ELISA (detecting antibodies against PfAMA1-DiCo1, DiCo2 or DiCo3 protein).

**Fig 6:**
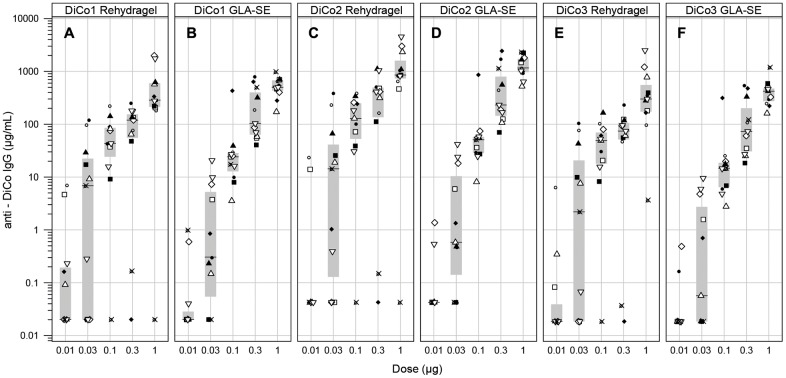
Seroconversion as a function of dose. Groups of 11 mice were immunized with indicated amounts of Drug Product formulated in either Rehydragel (Panels A, C and E) or GLA-SE (Panels B, D and F). On day 28 they were immunized again. Serum was collected on day 42 and ELISAs were performed using either PfAMA1 DiCo1 (Panels A,B), PfAMA1 DiCo2 (Panels C,D) or PfAMA1 DiCo3 (Panels E,F) as coating antigen. Individual mice within a treatment group are indicated by symbols, where the same symbol within a treatment groups represents the same animal for PfAMA1 DiCo1, PfAMA1 DiCo2 and PfAMA1 DiCo3, respectively.

As evident from Fig 6, seroconversion was dependent on dose. At 1 μg, the highest dose used, sero-conversion was high but a plateau was not yet reached. This dose was selected for the potency release assay (1 μg). To detect loss of potency in an early stage, the 0.1 μg dose was also included in the immune-potency assay.

Alhydrogel was chosen as the adjuvant to follow the potency of the Drug Product over time.

### Immunopotency as a function of real-time storage

**Fig 7:**
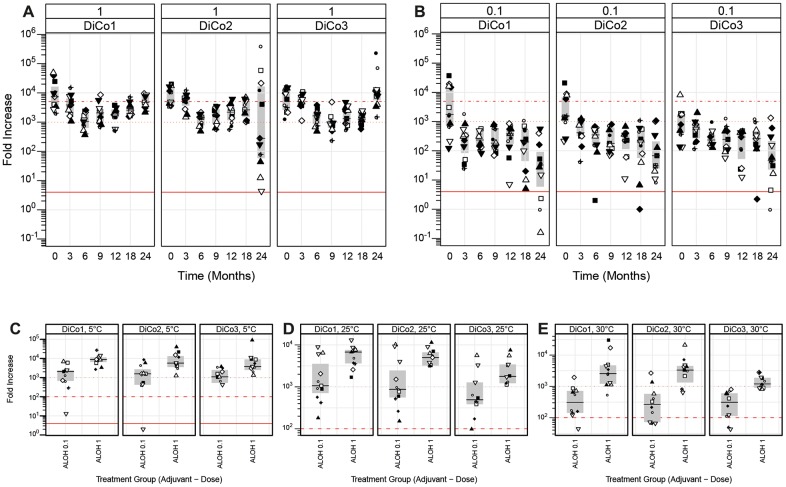
Potency of the Drug Product as a function of storage time. Panel A: Immunopotency of the drug product as a function of real-time storage time at -20°C for the high dose (1 μg Drug Product), formulated in Alhydrogel. The fold increase (seroconversion) is shown. Panel B: Immunopotency of the drug product as a function of real-time storage time at -20°C for the low dose (0.1 μg Drug Product), formulated in Alhydrogel. The fold increase (seroconversion) is shown. Panel C: Immunopotency of the formulated PfAMA1-DiCo vaccine (formulated with Alhydrogel) after storage for 24 hours at 5°C. Both dosages are shown. Panel D: Immunopotency of the PfAMA1-DiCo vaccine (formulated with Alhydrogel) after storage for 24 hours at 25°C. Both dosages are shown. Panel E: Immunopotency of the PfAMA1-DiCo Drug Product after storage for seven days at 30°C (before formulation). Immunopotency was measured as described in the Material and Methods section. Boxes indicate medians (middle) and quartile ranges (bottom and top).

At release, 11 mice were dosed subcutaneously with 1 μg PfAMA-DiCo Drug Product formulated with Alhydrogel or GLA-SE. At day 28 this was repeated. Blood sampling was performed at day 0 and 42. A more than 1000-fold increase in anti-DiCo 1, 2 or 3 specific IgG titer was observed in the sera of day 0 versus that of day 42 (For the Alhydrogel data, see Fig 7A, T = 0 values; for GLA-SE: data not shown).

As part of the stability test, at each time point, 11 mice were immunized with either 0.1 or 1 μg of the Drug Product, respectively, adjuvanted with Alhydrogel. Fig 7A and 7B show the immune potency profile over real-time, presented as the fold-increase between the Day 0 titre and the Day 42 titre. The actual titres can be found in the supplementary data (S2 Data).

The acceptance criterion for the potency, defined as a 4-fold increase in titer for the 1 μg dose (at day 42 vs day 0 of a potency assay time point), was met over the full 24-month time interval (Fig 7A). As expected, the 0.1 μg dose gave significantly lower antibody responses when compared to the 1.0 μg dose. Still, all mice seroconverted when the 0.1 μg dose was injected (compare Fig 7A and 7B).

An accelerated stability study, in which the Drug Product was stored for 7 days at 30°C showed that more than 80% of the mice immunized with either 0.1 or 1 μg AMA DiCo Drug Product seroconverted (Fig 7E).

### Additional exploratory assays

#### Conformational integrity of PfAMA1-DiCo Drug Product

A Gel shift assay, performed in analogy with those on the Drug Substances, essentially showed that the Drug Product quantitatively bound to the reduction-sensitive monoclonal antibody 4G2 (Fig 4). Moreover, determination of the amount of free sulfhydryls (free cysteines) in the Drug Product showed that these are virtually absent (<0.05 mol/mol) (data not shown).

#### Short-term stability in the presence of adjuvants

**Fig 8:**
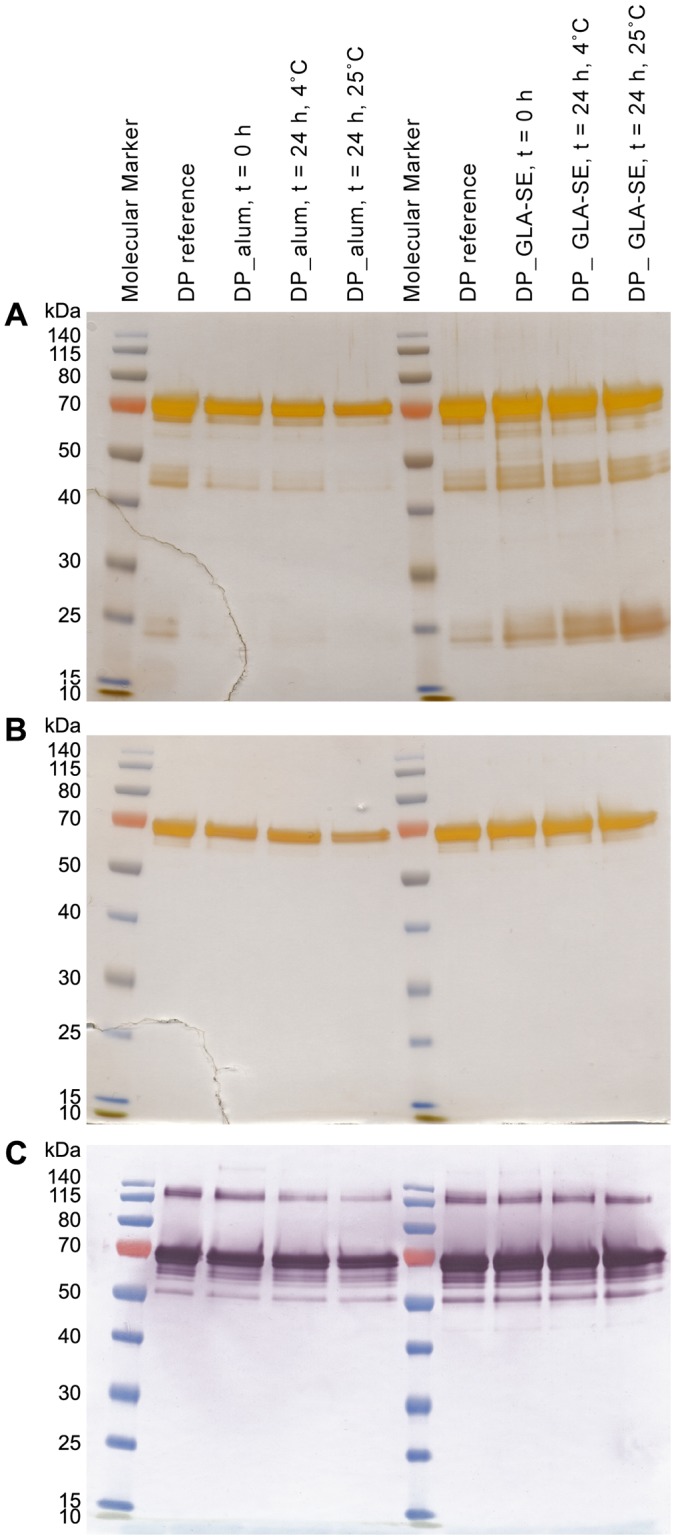
Short time stability of the Drug Product in the presence of adjuvants. Drug Product (DP) was formulated with either Alhydrogel (alum) or GLA-SE. Formulae were broken either directly after formulation (0 hours) or after 24 hours of storage at 4°C or at 25°C. Samples were analysed with SDS-PAGE, both reduced/silver staining (panel A) and non-reduced/silver staining (panel B) and by (non-reduced) western-blot with the monoclonal antibody 4G2 (panel C).

For the clinical phase I study, bedside mixing was the intended methodology. To test whether the vaccine is stable in formulated form in the refrigerator or at room temperature after overnight storage, the anticipated realistic maximal storage time, the Drug Product was mixed with the adjuvants to-be-used in the clinic and stored at 5°C and 25°C for 24 hours. Immunopotency assays results indicated that the biological activity was retained after 24 hours incubation at both temperatures (Fig 7C and 7D). Additionally, the formulas were broken and the proteins were analysed by several assays including reduced and non-reduced SDS-PAGE (Fig 8). These assays confirmed the stability of the formulated vaccine for at least 24h at both temperatures. ELISA assays also indicated more than 97.5% level of adsorption of the PfAMA1-DiCo Drug Product onto the aluminium hydroxide surface, which remained constant after 24h incubation. Particles size and zeta potential measurements of the Drug Product formulated with GLA-SE indicated the compatibility and stability of the formulation during the anticipated storage time (data not shown).

### Pharmacotoxicity

The toxicity assessment of the PfAMA1-AMA1-DiCo Drug Product, as well as toxicity of adjuvants alone and in combination with antigens, was done in a dedicated stand-alone repeated dose toxicity study in rabbits. Groups of 16 New Zealand Albino White rabbits, 8 of each sex, were immunized intramuscularly at days 1, 15, 29 and 43. Group 1 received saline, Group 2 received GLA-SE, Group 3 received PfAMA1-DiCo Drug Product, Group 4 received GLA-SE adjuvanted PfAMA1 DiCo Drug Product and Group 5 received Alhydrogel adjuvanted PfAMA1-DiCo Product.

On day 45 (5 animals of the main groups) or day 65 (3 animals of the recovery groups), all animals treated with PfAMA1-DiCo Drug Product (Groups 3, 4 and 5) showed PfAMA1-DiCo1, DiCo2 and DiCo3 specific antibodies (more than 1000-fold average increase) with no clear difference in response between the three PfAMA1-DiCo proteins. Pre-test sera and sera from animals of Groups 1 and 2 did not show reactivity with any PfAMA1-DiCo protein.

Addition of adjuvant (GLA-SE or Alhydrogel) to PfAMA1-DiCo Drug Product resulted in an approximately one- to eight-fold increase in PfAMA1-DiCo1, -DiCo2 and -DiCo3 specific antibodies on day 45 or 65. On day 65, the level of PfAMA1-DiCo1, -DiCo2 and -DiCo3 specific antibodies was higher for the Alhydrogel adjuvanted PfAMA1-DiCo Drug Product.

No treatment-related mortality, nor any toxicologically relevant changes in clinical signs and skin irritation, body weight, food consumption, ophthalmoscopic parameters, rectal body temperature, clinical biochemistry and organ weights were noted.

Treatment-related findings consisted of slight increases in fibrinogen in Group 2 (GLA-SE) and Group 4 (PfAMA1-DiCo + GLA-SE) two days after each vaccination and in Group 5 (PfAMA1-DiCo + Alhydrogel) two days after the second vaccination, and minor changes in protein levels.

Following the three-week recovery period, there were few findings in the injection sites or associated lymph nodes of Groups 1, 2 and 3. Persistent inflammatory and lymph node responses in Groups 4 and 5 were associated with the presence of granular foreign material. The findings indicate that the combination of PfAMA1-DiCo Drug Product plus either of the adjuvants produced the most tissue response and that these combinations were the most persistent in the tissues.

All observed macroscopic and microscopic findings consisted of a local low-grade inflammatory response with no meaningful frank tissue damage and with resolution of the response three weeks after the last dose. These findings were not considered to be adverse.

## Discussion

The production process for *Pichia*-produced PfAMA1 DiCo proteins as well as the fundamental downstream process unit operations were successfully transferred from the first generation PfAMA1 GMP processes, with minor modifications. The three PfAMA1-DiCo proteins differ from the first generation PfAMA1 FVO sequence between 9 and 25 amino acids [15], but these changes do obviously not have a dramatic effect on the expression/production levels. Also the basic purification scheme developed for the PfAMA1 FVO vaccine candidate could be implemented without major changes.

As mentioned earlier, an issue of particular importance was the stability of the Drug Substances. This was mended by changing the composition of the fermentation medium. Both the basal salts' concentration and the copper and cobalt ion concentration in the trace salts solution were reduced. Also, in the final process, biomass concentration was not ramped up to what is feasible with *Pichia*. The in-process-control acceptance criterium for the start of the induction was an OD_600_ > 200 (induction was started between OD_600_ of 220 to 240 in the three GMP runs) and a relatively short induction time of less than 24 h was used. The culture was adapted to methanol using 2 to 3 pulses of 0.1–0.25% (v/v) methanol and subsequently on-line methanol control at a concentration of 0.07% (v/v) was applied for 12–16 h. As an acceptance criterium for the bulk harvest, an OD_600_ >200 was set. Fresh weight and dry weight of the biomass was measured in the bulk harvest for information only, values were between 200 and 225 g/L for fresh weight and 55 g/L for dry weight in the three GMP runs.

Using this strategy, the stability profile of the proteins was improved, but the yield of the PfAMA1-DiCo proteins in the supernatant was comparatively low, in the range of 50–100 mg/L.

The purification of the PfAMA1 DiCo proteins was very similar to that of PfAMA1 FVO [17].

The initial capture step for the PfAMA1 DiCo proteins was IMAC, using Cu^2+^ as the ligand. The efficiency of this step was estimated to be close to 30% of the total amount of the DiCo-related protein in the supernatant (Table 1). SDS-PAGE analysis of IMAC samples showed that by far the largest proportion of the product binds to the Cu^2+^-charged matrix. However, washing at a low imidazol concentration eluted a large amount of protein that contained at least 8 major proteins including one at the size expected for the product (data not shown). This explains the loss of 30–50% of the DiCo-related proteins in the fermentation supernatant (Table 1). In the elution peak, obtained after application of 125 mM imidazole, there was only one major band at the expected size for the product that constituted approximately 90% of the eluate fraction. It has been observed that PfAMA1 proteins devoid of prodomain do not bind strongly to Cu-activated IMAC resin (B.W. Faber, unpublished observation). This, however, does not explain why full-length PfAMA1 DiCo proteins (same size bands on SDS-PAGE) bind less strong to the Cu-IMAC resin and can be washed away. However, as the yield and purity after this step was sufficient for clinical phase I, it was decided not to pursue further optimization of the purification procedure with respect to yield. The IMAC chromatograms and fractions were very similar for all three DiCos.

The HIC step that was included in the original protocol to remove *Pichia*-derived pigments [17] was also used in this protocol, although the IMAC eluate was much less coloured in the low-salt media that was used. An additional diafiltration step with a cut-off of 30 kDa was introduced in the purification procedure of PfAMA1 DiCo, to remove a 20 kDa product-related impurity that occurred after scale-up. This step essentially led to complete removal of the 20 kDa impurity, increasing the purity of the product to over 95% (Table 1). Finally, a gel filtration step was performed, basically to exchange the protein into a buffer suitable for storage.

SE-Chromatography showed that the individual PfAMA1-DiCo proteins (Drug Substances) were highly pure (97, 98 and 98%, for PfAMA1 DiCo1, DiCo2 and DiCo3 protein, respectively). Product-related impurities, mainly fragments or truncated DiCos accounted for the rest of the detectable proteinaceous impurities, reaching close to 100% for product and product-related impurities. Process-related impurity levels for copper, methanol and imidazole were analysed but not detectable or (far) below the set criteria in the bulk drug substance. Mass spectrometric analysis of SDS-PAGE bands showed that there was only one weak band in the SDS-PAGE gel that did not show product related peptides (PfAMA1 DiCo2 protein). However, these peptides were also not identified as being derived from *Pichia pastoris*.

All three PfAMA1-DiCo proteins are truncated at their N-terminal end, for approximately 40–50%. The N-terminal amino acids of the truncated proteins show that cleavage has occurred between Lys34 and Ser35. Cleavage at the domain II loop (R376-S377), illustrated by reducing the samples in the SDS-PAGE, was similar for the 3 PfAMA1-DiCo proteins. The cleaved fractions (Fig 2A) were not higher than those observed for PfAMA1 FVO [17] and lower than those observed for PfAMA1 3D7 [43].

The stability studies showed that the protease responsible for cleaving was removed efficiently during the purification procedure, as the level of cleavage, either in domain 2 or in the prodomain, did not increase upon storage. We have previously shown that the initial IMAC-step is crucial in that respect [44].

The apparent size at which the PfAMA1-DiCos run in reduced SDS-PAGE is closer to 70 kDa than to 59–60 kDa, the theoretical mass of the proteins. MS-analysis on pre-clinical material confirmed that the size of the proteins was indeed close to the theoretical values. The same discrepancy between the MS data and the apparent size on the SDS-PAGE was observed during the development and GMP-production of PfAMA1 FVO. Mass spectrometric analysis of undigested PfAMA1 FVO also showed a mass of approximately 59 kDa [17].

The specifications for the Drug Substances were set at the minimal acceptable levels as required by the European Medicines Agency (www.ema.europe.eu) (in the case of residual DNA, host cell protein and endotoxin) for early phase clinical trial, or at the detection limit for the method used (in the case of copper, methanol and imidazole). The commercially available host cell protein (HCP) assay kit for Pichia proteins showed cross-reactivity with the PfAMA1-DiCo proteins. This was unexpected because the kit was used during the PfAMA1 FVO GMP campaign and no cross-reactivity was observed. As the PfAMA1 DiCo proteins are very similar to the PfAMA1 FVO protein, batch-to-batch variation of the kit may be the reason for the observed cross-reactivity. Replacement of the ELISA based HCP-assay was not easy, because of its high sensitivity and specificity. The combination of silver-stained SDS-PAGE, western blotting and mass spectrometric data led to the conclusion that the residual host cell protein was well below 1%, the level of acceptance.

Preliminary stability studies with the Drug Substances revealed that dimers and aggregates were formed in the Drug Substances when stored in 0.1 mL aliquots in 2 mL PolyPropylene cryovials, while these were not observed in the Drug Substances stored in Flexboy bags. Therefore, stability studies of the GMP-grade purified bulk was carried out in 5 mL Flexboy bags to ensure as good as possible identical storage conditions of the bulk material and stability study aliquots, illustrating the importance of the ICH guidelines that state that “stability studies material should be stored in containers which properly represent the actual holding containers”.

Lyophilization is normally done to improve the stability of a drug product over time. This was indeed achieved for the Drug Product. It was however unexpected to observe that the experimental lyophilized Drug Product formulation that seemingly had the worst characteristics with respect to visual inspection (i.e. cake formation) had a very good recovery profile with regard to the main peak, and levels of dimers, multimers and by-products. Although the recovery profile characteristics for the experimentally formulated trehalose-containing excipients were also good (as well as the cake), the sucrose containing excipient was chosen because of the good stability profile that was obtained before (15).

All standard release assays for an investigational medicinal product such as appearance, appearance after dissolution, moisture content, sub-visible particles and sterility, were met by the PfAMA1-DiCo Drug Product. Furthermore, pH, endotoxin levels and protein content were measured and found to be within the predetermined criteria. The abnormal toxicity test was also passed. The biological activity of the PfAMA1-DiCo was demonstrated in immunopotency release assays where all mice immunised with PfAMA DiCo Drug Product formulated with Alhydrogel.

Determination of the amount of the individual PfAMA1-DiCos in the Drug Product was a challenge. First of all, the sandwich-ELISA that was developed for the PfAMA1 FVO product [17], using 4G2 as coating antigen and PfAMA1-DiCo-specific rabbit sera for detection was tried. This did not give satisfactory results as it turned out that there were subtle differences in the affinity of the PfAMA1-DiCo proteins for 4G2, leading to inconsistent results. As a second possibility, a competition ELISA was set up. It turned out that this was an adequate method to determine the presence of the individual PfAMA1-DiCo proteins in the mixture and the assay was selected as the release assay. However, the relatively high variance in the measurements (see Table 3) will not allow detection of selective deterioration of one of the components in the mixture. The assay is also quite laborious and time-consuming and requires a skilled operator. Moreover, obtaining the PfAMA1-DiCo specific reagentia (specificities of over 95%) is tedious. Therefore, although the competition ELISA methodology was useful at this point in time, for the future development of this potential vaccine, replacing this assay with a more reliable, less complex assay would be advisable.

We already started to explore this by setting up a Reversed Phase (RP)-HPLC assay. Given the strong resemblance of the proteins, as well as the heterogeneity in the N-terminal region, it was a challenge to find the right column and elution profiles to separate the proteins to such extent that quantification of the individual components was possible (Fig 5). The HPLC assay was used during the stability study, together with SPR and competition ELISA to determine possible deterioration of individual DiCo proteins in the Drug Product, believed to be important for the efficacy of the vaccine [15].

The concentration of PfAMA1 DiCo proteins in the Drug Product determined by μBCA (as total protein) and the concentrations of the individual DiCo proteins (sum of PfAMA1 DiCo1- PfAMA1 DiCo2 and PfAMA1 DiCo3 proteins), as determined by competition ELISA, were consistent. SPR-based calibration-free active concentration determination was used as an alternative method to determine the PfAMA1-DiCo protein content. The concentration determined (50.4 mg/mL) was 2-fold lower than expected (as determined by μBCA and competition ELISA). The SPR-based assay was performed on surface, featuring the monoclonal antibody 4G2, which recognizes an epitope located at the base of the flexible loop in domain II [45]. The lower concentration observed in the CFCA experiment that (unlike μBCA and ELISA) relies on rapid binding of the PfAMA1 DiCo proteins to the 4G2 surface may be explained by assuming different conformations of the loop region in domain II, possibly induced by the partial cleavage (nick) that has been demonstrated for about 50% of the PfAMA1 DiCo proteins. Assuming two conformations, we speculate that one conformation readily binds to 4G2, while the other variant only binds strongly to 4G2 after a contact-induced conformational change that takes time, which is not available in the SPR-assay set-up. This hypothesis of an induced conformational change is supported by our observation that the PfAMA1 DiCo proteins quantitatively bound to 4G2 in a mobility-shift assay (Fig 4), additionally it is known that the 4G2-binding region is indeed naturally flexible and a subject of conformational changes induced by the binding of its natural interaction partner PfRON2 [46].

Despite the systematic 2-fold too low values that were obtained, we believe the CFCA method represents an attractive alternative assay to address the concentration of the Drug Product, as it will depend much less on the operator, will be easy to reproduce and to repeat and there is a potential to "improve" the assay using different monoclonal antibodies recognizing epitopes that are not subject to (induced) conformational changes.

The Drug Product bound to the mAb 4G2 both on western blot and (quantitatively) in solution (Figs 2C and 4), showing the intactness of the discontinuous, reduction-sensitive 4G2-epitope in the proteins produced (see discussion above). This is taken as a surrogate measure of conformational integrity. The fact that the proteins elicit functional antibodies [15] confirms this view. Moreover, free sulfhydryls were below the detection limit of the method, indicative for intact disulphide bridges, again suggesting proper conformation of the proteins. Proper folding is considered to be important, as it has been shown that unfolded PfAMA1 does not induce functional antibodies [41].

All release assays were performed at each time point of the real time stability study, apart from the sub-visible particle counting, the sterility, endotoxin level and abnormal toxicity assays. Sterility will be repeated at the last time point and sub-visible particles counting are performed with a minimal frequency of 6 months. The accelerated stability study (7 days 30°C) predicted that the Drug Product would have a good stability profile, which was confirmed by the real time stability studies that reveal that up to 24 months there are no signs of the Drug Product deterioration (Table 3).

The potency release assay was established on basis of a dose-response curve in mice (Fig 6). For the release assay the 1 μg dose was selected. The 1 μg dose is in the linear part of the dose-response curce, and consistently induces high titers in over 80% of the immunized mice. Still, as evident from Fig 6, some animals do not respond. For the 0.1 μg dose, the number of non-responding animals as well as the spread in the FI-values is higher than for the 1 μg dose (Fig 6). For this reason the 1 μg dose was chosen in the release assay, but, as it was realized that this was not a very sensitive way to monitor loss of potency, the 0.1 μg dose was included in the studies to get a more realistic idea about the potency over time.

Fig 7 shows that there are statistically significant differences in FI for numerous time points, dosages and antigens (For details see S2 Data). This suggests a loss of potency of the drug product over time. Also the absolute titres at D42 show a number of statistically significant differences, especially at the T18 and T24 time points, if compared to the T0 value (S2 Data). However, the T = 36 potency data, either presented as FI or as absolute Day42 titres do not significantly differ from the T0 value, not for the 1 μg nore for the 0.1 μg dose (S2 Data), hinting to the possibility that the observed differences may have been caused by experimental issues connected to the use of the mouse model. The minor deterioration of the Drug Product observed in the other stability tests (f.e. Size Exclusion Chromatography) warrants this conclusion. However, on basis of the above, it still cannot be excluded that the potency went down at first and came back to the original levels in a later stage. This conclusion in itself warrants development of a more reliable, robust potency release assay for future use.

The dose levels (50 μg/0.5 mL), formulation, immunisation schedule (days 1, 15, 29 and 43), frequency (at a decreased interval), the route of administration (IM), clinical observations, laboratory investigations, macroscopic examination and histopathology used in the pharmacotoxicity study were chosen on basis of the already existing proposal for the clinical evaluation of the PfAMA1-DiCo vaccine.

Based on the results obtained in the pharmacotoxicology GLP study, it can be concluded that four intramuscular administrations with GLA-SE adjuvant, PfAMA1-DiCo Drug Product, GLA-SE adjuvanted PfAMA1-DiCo Drug Product or Alhydrogel adjuvanted PfAMA1-DiCo Drug Product in rabbits were well tolerated and did not induce any adverse effects in terms of systemic and local toxicity.

With this study we show that production, down-stream processing and quality assessment of three closely related proteins is achievable with identical procedures and release assays as anticipated before the start of the project: the full process was developed for the PfAMA1-DiCo1 protein and subsequently applied on the other two proteins, already far in the process (technical runs). This concept is likely to be transferable to antigens with a similar polymorphism profile, such as influenza virus hemagglutinin.

Given the Drug Product characterization profile, the positive toxicology study outcome and the acceptable stability profile, the PfAMA1-DiCo Drug Product has entered clinical evaluation (phase Ia/Ib) in early 2014 (ClinicalTrials.gov Identifier: NCT02014727) and is now near to completion.

## Supporting Information

S1 Data Determination of Host Cell Proteins (HCP) in PfAMA1 DiCo Drug Substances. (PDF)

S2 Data IgG titers and statistical analysis of the potency data. (PDF)
