# Supplementary material for: Production, Quality Control, Stability and Pharmacotoxicity of a Malaria Vaccine Comprising Three Highly Similar PfAMA1 Protein Molecules to Overcome Antigenic Variation
Source: PLoS One. 2016 Oct 3;11(10):e0164053. doi: 10.1371/journal.pone.0164053 (PMC5047445; doi:10.1371/journal.pone.0164053)
Supplement: S1 Data — (PDF) [file pone.0164053.s001.pdf]

Supplementary data 1. Determination of Host Cell Proteins (HCP) in PfAMA1 DiCo Drug Substances

In order to quantify HCP levels in the PfAMA1 DiCo API batches, the commercially available Pichia pastoris HCP ELISA-kit (Cygnus Technologies, Southport, USA) was applied.

This kit is regarded as useful for APIs in the process development phase. However, application of the Kit to the PfAMA1 DiCo proteins to determine HCP levels was unsatisfactory. Linearity could not be proven and the HCP values for the different dilutions were not consistent. As these results may be explained by assuming cross-reactivity of the antibodies provided in the Cygnus HCP ELISA-kit with the PfAMA1 DiCo proteins, western blot analysis using anti-sera from the Cygnus kit on DiCo protein was performed, essentially confirming the cross-reactivity (figure S1).

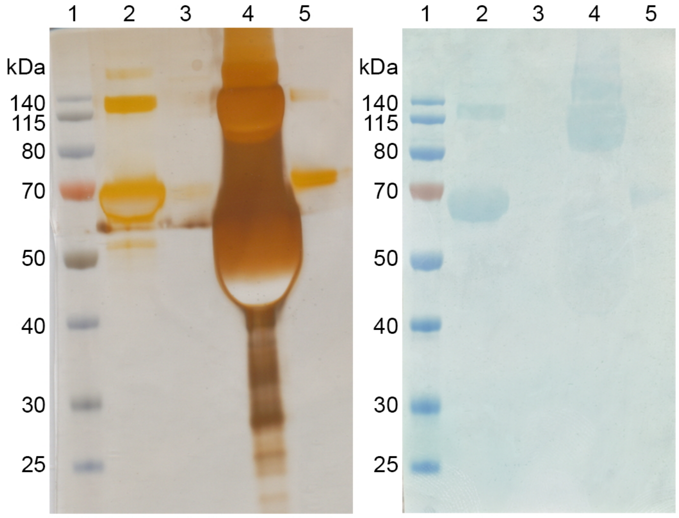

Figure S1. Cross-reactivity of anti-HCP antibodies with PfAMA1 DiCo protein. Left-hand panel: SDS-PAGE /silver-staining; Right hand panel: Western analysis with anti-HCP antibodies, HRP conjugated. Lane 1 Molecular marker; lane 2 PfAMA1 DiCo1; lane 3, empty; lane 4, *Pichia pastoris* protein lysate; lane 5, PfAMA1 DiCo1 (low concentration).

Subsequent trials to remove the DiCo proteins specifically by binding them to the 4G2 antibody before analyzing HCPs with the ELISA were not successful. Technical support from Cygnus Technologies

suggested to extend the dilution range of the PfAMA1 DiCo proteins in the ELISA to obtain dilutional linearity, but even a dilution to 1:64,000 could not solve the problems.

In conclusion, the reliability of the ELISA could not be proven at this stage in development for the DiCo batches and an alternative was pursued for the determination of the HCP in the API batches.

Determination of HCP using SDS-PAGE in combination with silver staining.

A method was chosen based on SDS-PAGE and silver staining. Subsequently, the silver stained bands were recognized and confirmed by specific antibodies in Western Blot. This method allows for a sensitivity limit of less than 1%, as required by the regulatory authorities. Visual inspection of the gels and blots revealed that all bands visible in silver staining could be linked to immunostaining of Western blots with the DiCo-specific 4G2 antibody (Figure S2). This antibody did not cross-react with with *P. pastoris* HCPs from the Cygnus ELISA-kit in Western Blot (data not shown).

Confirmation of HCP content by mass spectrometry

For additional supportive data, mass spectrometric analysis of the bands from SDS-PAGE under both reducing and non-reducing conditions was conducted. Masses of the extracted peptides were compared to the MASCOT database and to a library comprized of tryptic digests of the DiCo PfAMA1 proteins. Of the 28 bands excised from the gels, tryptically digested and analysed by MS-MS analysis (Figure S3), 23 of these bands could be attributed to the relevant DiCo protein unambiguously (Table S1). Of the 5 bands that were not identified, 4 could also not be identified using a large protein database. The last peptide that could not be attributed to a DiCo peptide using the DiCo database was identified as a microbial tryptophanase, using the general protein database, not related to *Pichia* nor human. The MS results for this band was not of high significance.

Two peptides that were attributed to DiCo AMA1 using the DiCo database were attributed to a non-AMA-1 related protein (histone H2B and cytochrome P450 monooxygenase, respectively) using the

general protein database search. It should be noted that the MS results for these two bands were not of high significance and a BLAST search of the sequences revealed that the peptides were not listed in connection with *P. pastoris* – proteins.

Taken together, these findings justify the statement that HCP content in the DiCo preparations is <1%.

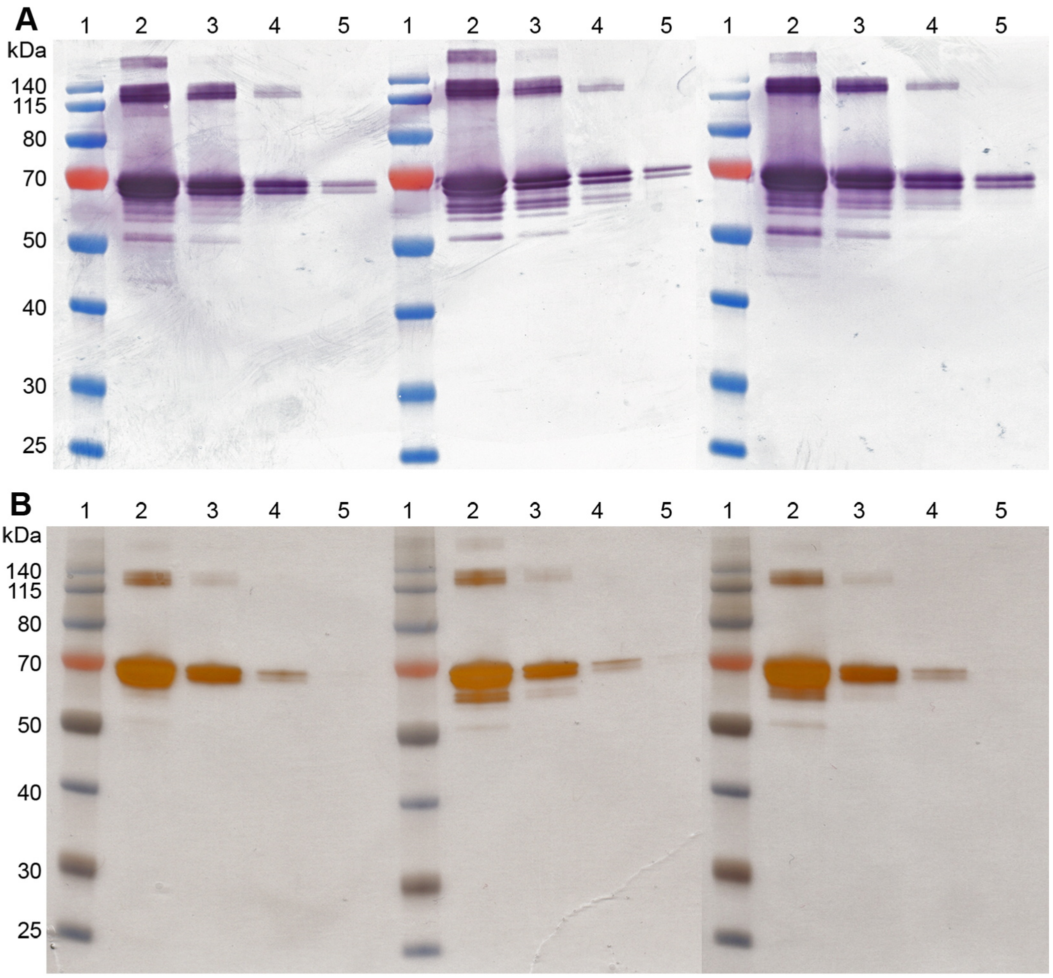

Figure S2. A) Western blots for PfAMA1 DiCo proteins using the mAb 4G2. Left hand panel, PfAMA1 DiCo1; Middle panel, PfAMA1 DiCo2; Left hand panel, PfAMA1 DiCo3. Lane 1, Marker, lane 2, 2 µg; lane 3, 0.4 µg; lane 4, 80 ng; lane 5, 16 ng. B) Silver-stained SDS-PAGE gels for PfAMA1 DiCo proteins. Left hand panel, PfAMA1 DiCo1; Middle panel, PfAMA1 DiCo2; Left hand panel, PfAMA1 DiCo3. Lane 1, Marker, lane 2, 2 µg; lane 3, 0.4 µg; lane 4, 80 ng; lane 5, 16 ng. Samples were not reduced

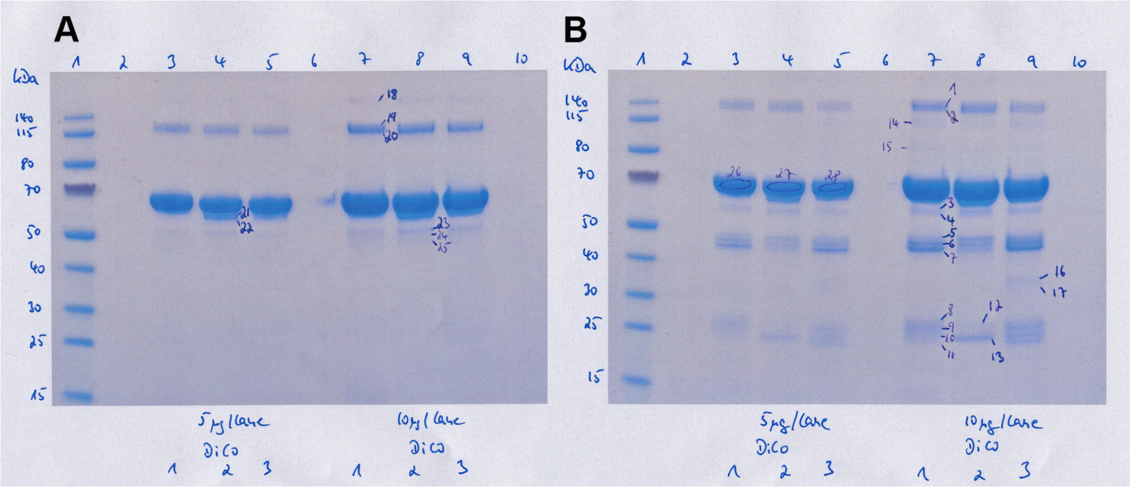

Figure S3. SDS-gels of reduced (A) and non-reduced (B) DiCo proteins (Drug Substance) with indicated bands that were analyzed with Mass Spectrometry. Numbers refer to the bands in Table S1. Panel A, (non-reduced samples) lane 1, Molecular marker; lane 2, empty; lane 3, DiCo1 5 µg; lane 4, DiCo2 5 µg; lane 5, DiCo3 5 µg; lane 6, empty, lane 7, DiCo1 10 µg, lane 8, DiCo2 10 µg; lane 9, DiCo3 10 µg, lane 10, empty. Panel B, (reduced samples) lane 1, Molecular marker; lane 2, empty; lane 3, DiCo1 5 µg; lane 4, DiCo2 5 µg; lane 5, DiCo3 5 µg; lane 6, empty, lane 7, DiCo1 10 µg, lane 8, DiCo2 10 µg; lane 9, DiCo3 10 µg, lane 10, empty.

Table S1. Mass spectrometric analysis of the gel bands depicted in Figure S3.

Band nr.	Protein Database Search (MASCOT)	DiCo Search
1	Apical membrane antigen 1 [Plasmodium falciparum]	DiCo_1
2	Apical membrane antigen 1 [Plasmodium falciparum]	DiCo_1 / DiCo_3
3	Keratin/ Apical membrane antigen 1 [Plasmodium falciparum]	DiCo_1
4	No result	DiCo_1
5	Keratin; Apical membrane antigen 1 [Plasmodium falciparum]	DiCo_1
6	Apical membrane antigen 1 [Plasmodium falciparum]	DiCo_1
7	Apical membrane antigen 1 [Plasmodium falciparum]	DiCo_1
8	No result	DiCo_1
9	Apical membrane antigen 1 [synthetic construct]	DiCo_1
10	Apical membrane antigen 1 [synthetic construct]	DiCo_1
11	Apical membrane antigen 1 [Plasmodium falciparum]	DiCo_1
12	No result	No result
13	Apical membrane antigen 1 [synthetic construct]	DiCo_2
14	No result	No result
15	Tryptophanase [Pasteurella dagmatis ATCC 43325]	No result
16	Apical membrane antigen 1 [Plasmodium falciparum]	DiCo_3
17	Apical membrane antigen 1 [Plasmodium falciparum]	DiCo_3
18	No result	No result
19	Apical membrane antigen 1 [synthetic construct]	DiCo_1
20	Apical membrane antigen 1 [synthetic construct]	DiCo_1
21	Apical membrane antigen 1 (Plasmodium falciparum]	DiCo_2
22	Histone H2B [Human]	DiCo_2
23	Cytochrome P450 monooxygenase [Nocardia farcinica IFM 10152]	DiCo_1/ DiCo_2
24	Apical membrane antigen 1 [synthetic construct]	DiCo_1/ DiCo_2
25	No result	No result
26	Apical membrane antigen 1 [Plasmodium falciparum]	DiCo_1
27	Apical membrane antigen 1 [synthetic construct]	DiCo_2
28	Apical membrane antigen 1 [Plasmodium falciparum]	DiCo_3
