# Supplementary material for: Production, Quality Control, Stability and Pharmacotoxicity of a Malaria Vaccine Comprising Three Highly Similar PfAMA1 Protein Molecules to Overcome Antigenic Variation
Source: PLoS One. 2016 Oct 3;11(10):e0164053. doi: 10.1371/journal.pone.0164053 (PMC5047445; doi:10.1371/journal.pone.0164053)
Supplement: S2 Data — (PDF) [file pone.0164053.s002.pdf]

Supplementary data 2. IgG titers and statistical analysis of the potency data

Fig S2.1 shows the amount of PfAMA1 specific antibodies, as determined in the sera from the mice immunized for the potency study at Day 0 and at Day 42, 14 days after the second immunization. The rightmost panel shows that ratio of the day 42 and the day 0 titres (See also Fig 7).

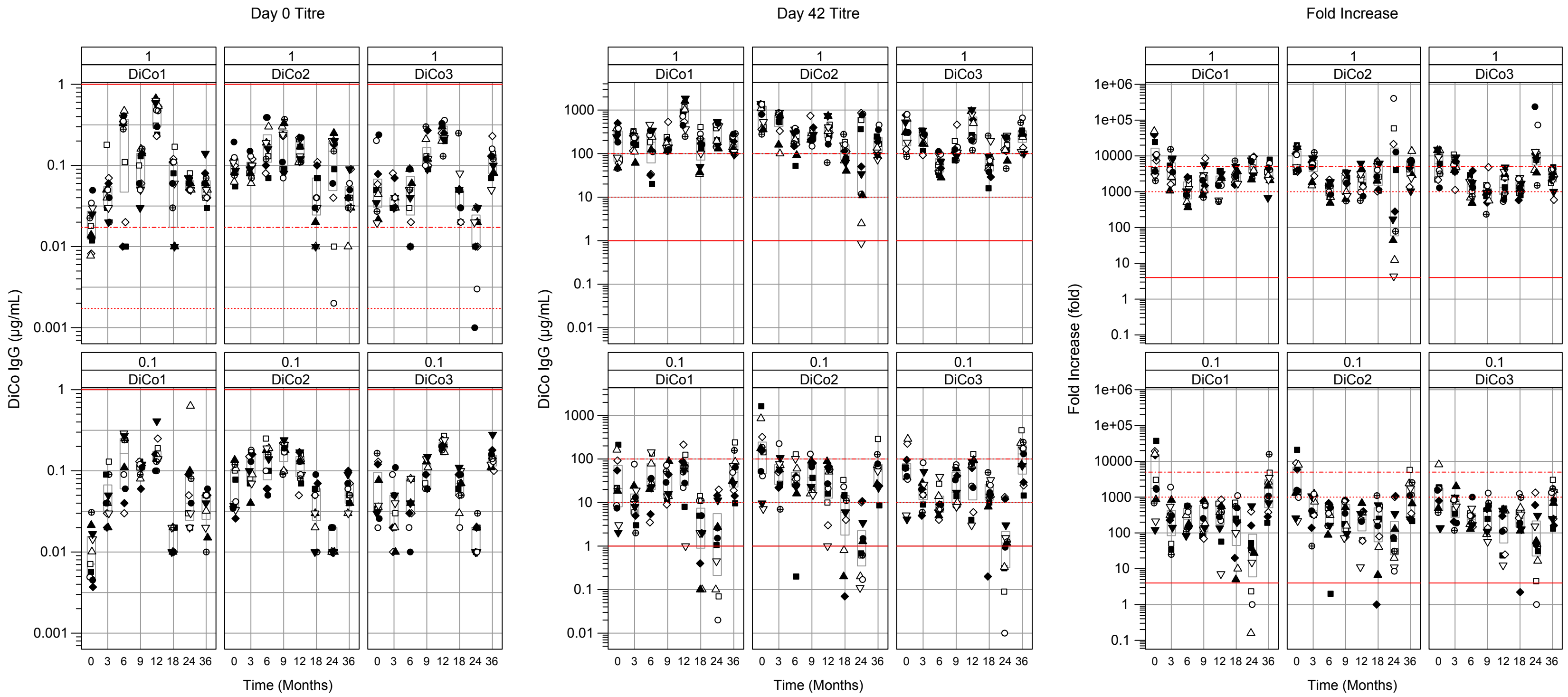

FigureS2.1 Absolute IgG titres (days 0 and 42) and Fold Increase as a function of storage time.
Values obtained for individual mice are indicated by the same symbols (per time point for DiCo 1-3 and IgG titres at days 0, 42 and the fold increase (value for day42/value for day0). Upper panels show the results for the 1 µg dose, lower panels for the 0.1 µg dose.
Boxes indicate medians (middle) and quartile ranges (bottom and top).

Fig S2.2 shows the statistical analysis assessing the differences between the potency data obtained at Day 0 (at the start of the stability study) and indicated time points (3, 6, 9, 12, 18, 24 and 36 months). The fold difference is shown. Upper panels show the 1 µg dose, lower panels the 0.1 µg dose.

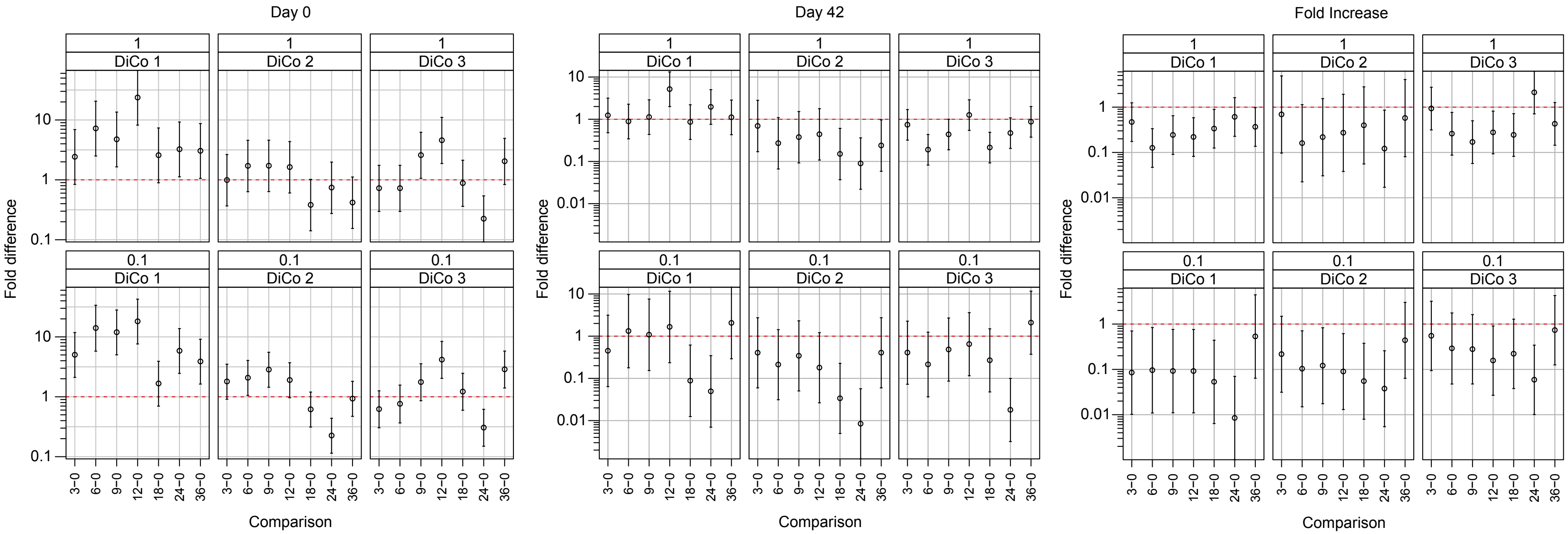

FigureS2.2. Fold differences between T = 0 and follow up time points, for the Day 0 titres (left hand panel), the Day 42 titres (middle panel) and the Fold Increase (right hand panel). Points depict fold differences between indicated time points and error bars depict 95% Confidence Intervals. If the error bar does not include unity (fold difference = 1) this implies that there is a statistically significant difference between this data point and the T = 0 value.

Statistical analysis

ANOVA was used to evaluate differences between the time points. IgG titres were log-transformed and post-hoc comparisons were performed using Tukey's Honest Significant Difference. Differences between T = 0 and the follow-up time points are expressed as ratio's with 95% Confidence Intervals, calculated from the Tukey HSD analysis.
